# Heme in pathophysiology: a matter of scavenging, metabolism and trafficking across cell membranes

**DOI:** 10.3389/fphar.2014.00061

**Published:** 2014-04-08

**Authors:** Deborah Chiabrando, Francesca Vinchi, Veronica Fiorito, Sonia Mercurio, Emanuela Tolosano

**Affiliations:** Department of Molecular Biotechnology and Health Sciences, Molecular Biotechnology Center, University of TurinTurin, Italy

**Keywords:** hemopexin, FLVCR1, FLVCR2, ABCG2, HCP1/PCFT, HO-1

## Abstract

Heme (iron-protoporphyrin IX) is an essential co-factor involved in multiple biological processes: oxygen transport and storage, electron transfer, drug and steroid metabolism, signal transduction, and micro RNA processing. However, excess free-heme is highly toxic due to its ability to promote oxidative stress and lipid peroxidation, thus leading to membrane injury and, ultimately, apoptosis. Thus, heme metabolism needs to be finely regulated. Intracellular heme amount is controlled at multiple levels: synthesis, utilization by hemoproteins, degradation and both intracellular and intercellular trafficking. This review focuses on recent findings highlighting the importance of controlling intracellular heme levels to counteract heme-induced oxidative stress. The contributions of heme scavenging from the extracellular environment, heme synthesis and incorporation into hemoproteins, heme catabolism and heme transport in maintaining adequate intracellular heme content are discussed. Particular attention is put on the recently described mechanisms of heme trafficking through the plasma membrane mediated by specific heme importers and exporters. Finally, the involvement of genes orchestrating heme metabolism in several pathological conditions is illustrated and new therapeutic approaches aimed at controlling heme metabolism are discussed.

## The “good” and the “bad” face of heme

### Heme as a prostetic group in hemoproteins

Heme is an iron-containing porphyrin of vital importance since it constitutes the prosthetic moiety of several hemoproteins. It can interact with different inactive apo-heme proteins giving rise to active hemoproteins, whose function is ultimately determined by the properties of the polypeptide bound to it (Dawson, 1988). Thanks to its ability to coordinate an iron atom inside its structure, heme is engaged in controlled oxido-reducing reactions that allow hemoproteins to work properly. For this reason, heme is involved in a multitude of crucial biological functions. In hemoglobin and myoglobin, it is used for oxygen transport and storage, respectively, whereas in cytochromes it is involved in electron transport, energy generation, and chemical transformation. In catalases and peroxidases, heme functions in hydrogen peroxide inactivation or activation, respectively, and in tryptophan pyrrolase, it catalyzes the oxidation of tryptophan (Kumar and Bandyopadhyay, 2005). Furthermore, heme is indispensable for a wide array of other important enzyme systems, such as cyclooxygenase and nitric-oxide synthase (Seed and Willoughby, 1997).

### Heme as a modulator of gene expression and cell proliferation/differentiation

Other than acting as a prostetic group in hemoproteins, heme itself may influence the expression of many genes. In non erythroid cells, heme regulates its own production by down-regulating heme biosynthesis at the level of the rate-limiting enzyme 5-aminolevulinic acid (ALA) synthase 1 (ALAS1) and by up-regulating its metabolism (Yamamoto et al., 1982). Conversely, in erythroid cells, heme acts as a positive feedback regulator for its synthesis and inhibits its degradation (Sassa, 1976; Rutherford and Harrison, 1979). Heme may control gene expression at almost all levels by regulating transcription, mRNA stability, splicing, protein synthesis, and post-translational modification (Ponka, 1999; Zhu et al., 1999). Genes coding for globins, heme biosynthetic enzymes, heme-oxygenase (HO)-1, ferroportin, cytochromes, myeloperoxidase, and transferrin receptor are all regulated by heme. Most of these genes are regulated via heme response elements (HREs) and the mammalian transcription repressor, Bach1 (Ogawa et al., 2001). Heme responsive elements (HREs) are located in enhancer regions of genes induced by heme itself. The minimal heme response element was identified as an extended activator protein 1 (AP-1, c-Fos/c-Jun family) binding site, TGCTGAGTCAT/C. In addition to the AP-1 heptad (TGAGTCA), this element also contains an interdigitated antioxidant response element (ARE), GCnnnGTCA. This motif resembles the binding site of the transcription factors of the sMaf family (Maf recognition element, MARE; consensus sequence: TGCTGAC(G)TCAGCA), which contains an ARE core for transcription factors of the NF-E2 family (NF-E2, Nrf1, Nrf2) (Inamdar et al., 1996). The transcription factor Bach1 interacts with proteins of the Maf-related family and the resulting heterodimer binds HRE elements, thus repressing gene transcription. Under conditions of intracellular heme accumulation, heme binds to Bach1, thus leading to a conformational change, a decrease in DNA binding activity and finally its removal from HREs (Marro et al., 2010). This allows Maf-Maf, Nrf2-Maf, and other activating heterodimers (Nrf2-AP-1) to occupy the HRE sites in gene promoters and leads to increased transcription of target genes.

In addition, heme regulates differentiation and proliferation of various cell types. It stimulates neuronal differentiation of mouse neuroblastoma cells (Ishii and Maniatis, 1978), erythroid differentiation of erythroleukemia cells (Granick and Sassa, 1978), formation of erythroid colonies in mouse as well as in human bone marrow cultures (Partanen et al., 1988; Abraham et al., 1991), differentiation of 3T3 fibroblasts into adipocytes (Chen and London, 1981), and it stimulates cell growth of cultured fibroblasts (Verger et al., 1983).

All together, these functions can be regarded as the “good face” of heme, without which many critical biological processes would not occur.

### Free heme toxicity

**Figure 1:**
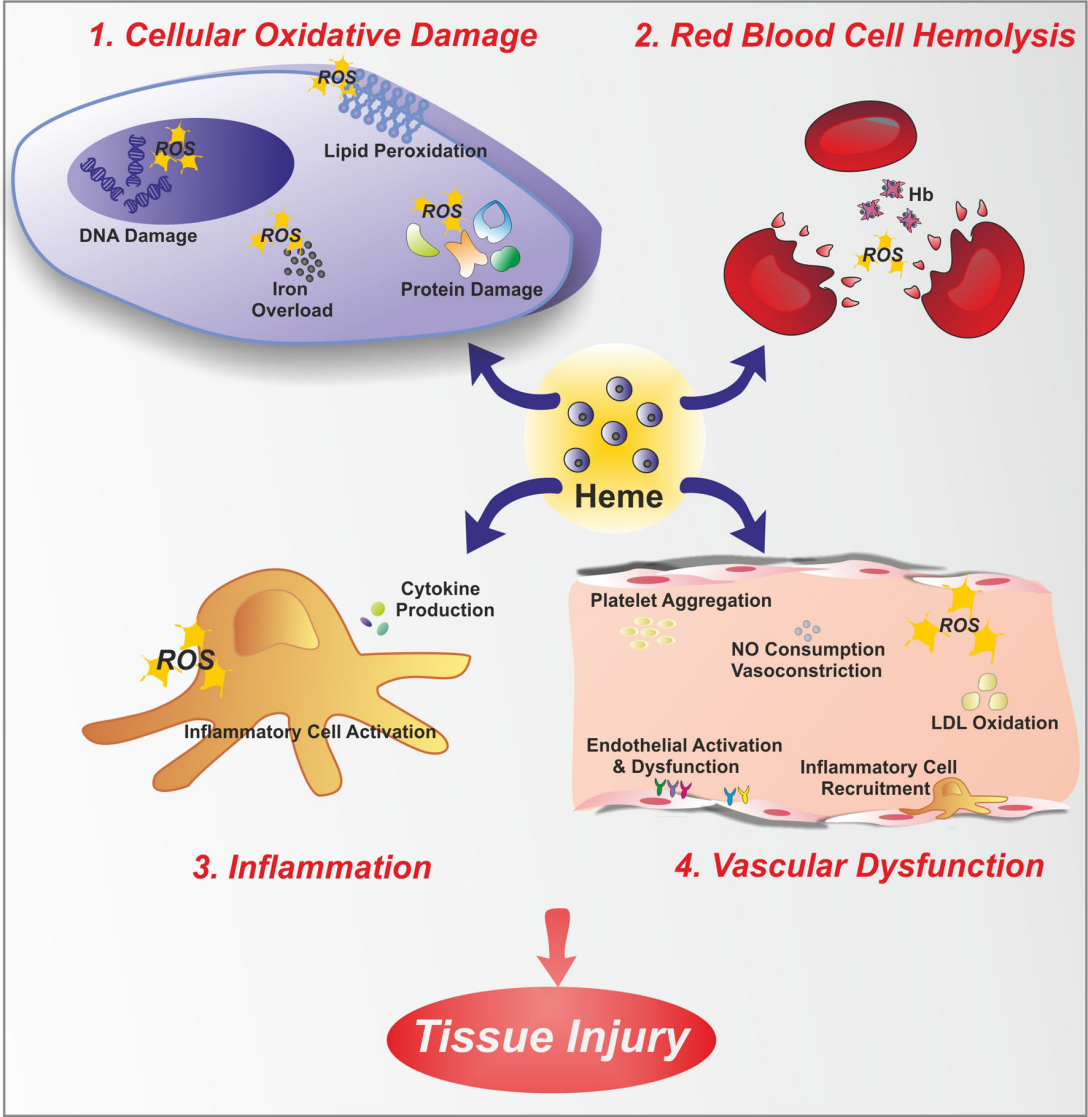
**Free heme toxicity**. Free heme has potentially toxic properties due to the catalytic active iron atom it coordinates. Here, toxic effects of heme are depicted. Heme causes cellular oxidative damage **(1)** by promoting ROS formation, lipid peroxidation, DNA and protein damage. Additionally, heme is a source of iron. Therefore, heme overload leads to intracellular accumulation of iron, with further ROS generation. Heme is a hemolytic agent **(2)**, since it intercalates red blood cell membrane, thus favoring cell rupture and further amplifying the hemolytic process. Heme promotes inflammation **(3)**, by stimulating inflammatory cell activation and cytokine production. Finally, heme causes endothelial dysfunction **(4)** by several mechanisms: increasing adhesion molecule expression and endothelial activation, promoting inflammatory cell recruitment and platelet aggregation, causing nitric oxide (NO) oxidative consumption and vasoconstriction, oxidizing low-density lipoprotein (LDL).

In contrast to the positive functions of heme, free heme excess can cause cell damage and tissue injury since heme catalyzes the formation of reactive oxygen species (ROS), resulting in oxidative stress. Heme that is not bound to proteins is considered the labile heme pool; this portion of heme is derived from newly synthesized heme that has not yet been incorporated into hemoproteins, or heme that has been released from hemoproteins under oxidative conditions. “Free heme” is an abundant source of redox-active iron that can participate in the Fenton reaction to produce toxic free hydroxyl radicals. ROS damage lipid membranes, proteins and nucleic acids, activate cell signaling pathways and oxidant-sensitive, proinflammatory transcription factors, alter protein expression, and perturb membrane channels (Vercellotti et al., 1994; Jeney et al., 2002). Heme toxicity is further exacerbated by its ability to intercalate into lipid membranes. Due to its lipophilic nature, heme may initially lodge within the hydrophobic phospholipid bilayer. Within this highly oxidizable matrix, iron catalyzes the oxidation of cell membrane and promotes the formation of cytotoxic lipid peroxide, which enhances membrane permeability, thus promoting cell lysis and death (Balla et al., 1991; Ryter and Tyrrell, 2000; Kumar and Bandyopadhyay, 2005; Tolosano et al., 2010). Additionally, heme is a potent hemolytic agent. It affects erythrocyte membrane stability as a result of ROS formation and oxidative membrane damage. Finally, heme is strongly pro-inflammatory since it induces the recruitment of leukocytes, platelets and red blood cells to the vascular endothelium, it oxidizes low-density lipoproteins and it consumes nitric oxide, thus impairing vascular function (Figure 1).

When intracellular heme accumulation occurs, heme is able to exert its pro-oxidant and cytotoxic action. The cellular free heme pool may increase after extracellular heme overload, increased heme synthesis, accelerated hemoprotein breakdown, impaired incorporation into apo-hemoproteins, or impaired HO activity, resulting in ROS formation, oxidative damage and cell injury. Several pathological conditions are associated with hemolysis or myolysis, and tissues can subsequently be exposed to large amounts of free heme (sickle cell anemia, thalassemia, malaria, paroxysomal nocturnal hemoglobinuria, etc.) (Gozzelino et al., 2010).

In summary, heme is a double-faced molecule: physiological amounts of heme act in gene regulation or as the functional group of hemoproteins, providing essential cellular functions, whereas excessive free heme levels result in oxidative stress and tissue injury. Therefore, the amount of free heme must be tightly controlled to maintain cellular homeostasis and avoid pathological conditions. To this purpose, mammals evolved several defense mechanisms to specifically counteract free heme-mediated oxidative stress and inflammation (Wagener et al., 2003; Gozzelino et al., 2010).

Here the mechanisms involved in the maintenance of heme homeostasis and in the control of heme levels are reviewed: the regulation of both extracellular and intracellular heme content is described, with particular emphasis on the emerging role of heme transporters.

## Regulation of extracellular heme content

Mammals are equipped with various systems able to prevent extracellular heme toxicity. Among them, a key function is covered by the soluble scavengers of free hemoglobin and heme, Haptoglobin (Hp) (Schaer and Buehler, 2013; Schaer et al., 2013a) and Hemopexin (Hx) (Tolosano et al., 2010), respectively.

**Figure 2:**
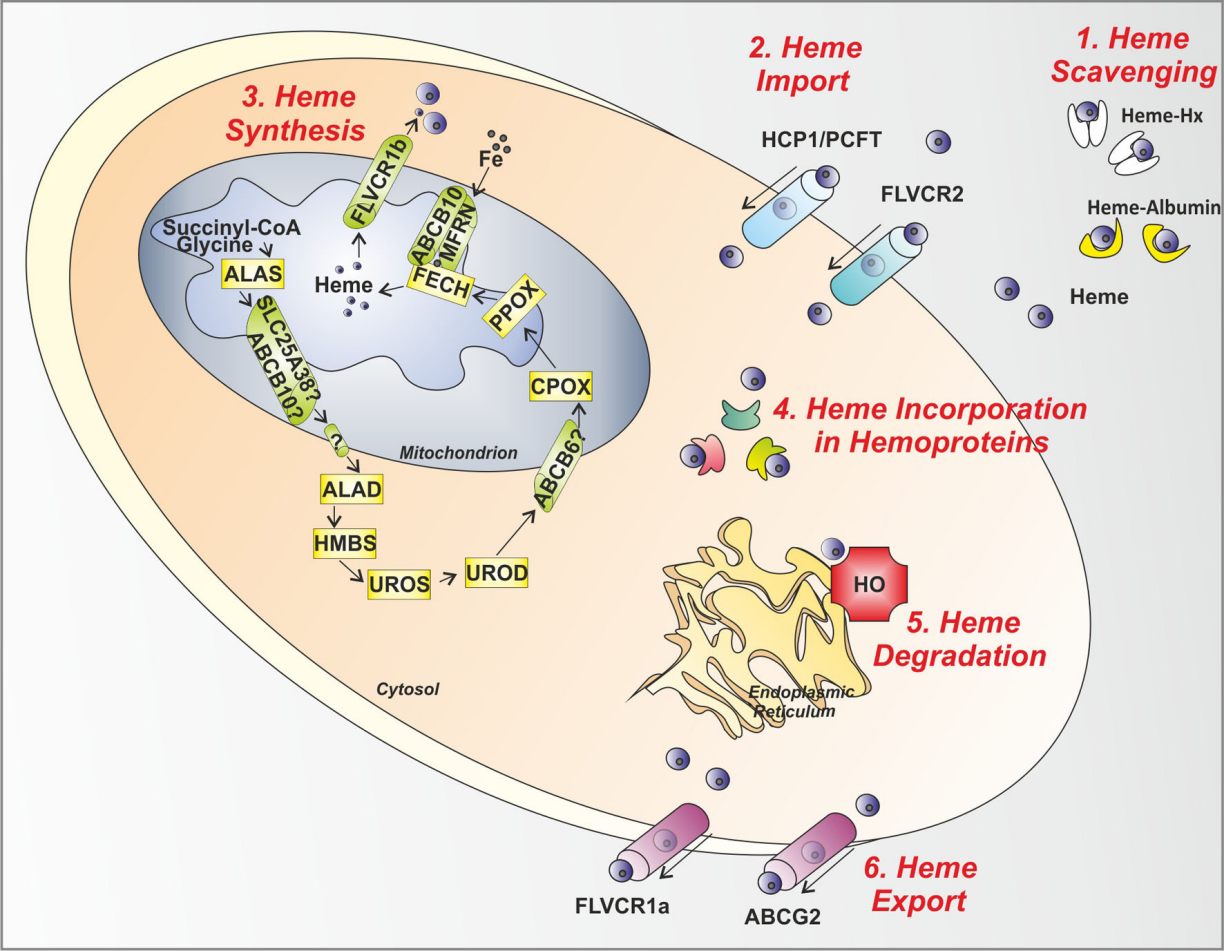
**Control steps in heme metabolism**. The main mechanisms involved in the control of heme levels outside, inside and across the cell are illustrated. **(1)** Heme scavenging: Circulating free heme toxicity is avoided thanks to the action of the scavenging proteins Hx and Albumin. **(2)** Heme Import: Heme might be imported inside the cell via the putative heme importers HCP1/PCFT and FLVCR2. **(3)** Heme Synthesis: in the mitochondrion and cytosol, the heme biosynthetic enzymes, starting from succinyl-CoA and glycine, give rise to heme. After synthesis, heme is exported out of the mitochondrion to the cytosol by the mitochondrial heme exporter FLVCR1b. **(4)** Heme Incorporation in Hemoproteins: once released in the cytosol, heme is inserted in apo-hemoproteins to allow the formation of functional hemoproteins. **(5)** Heme Degradation: in the endoplasmic reticulum, the heme degrading enzyme HO is responsible for heme degradation into iron (Fe), carbon monoxide and biliverdin. **(6)** Heme Export: the heme exporters FLVCR1a and ABCG2 regulate heme export out of the cell across the plasma membrane. ALAS, aminolevulinic acid synthase; SLC25A38, solute carrier family 25 member 38; ABCB10, ATP-binding cassette sub-family B member 10; ALAD, amino levulinic acid dehydratase; HMBS, hydroxymethylbilane synthase; UROS, uroporphyrinogen III synthase; UROD, uroporphyrinogen decarboxylase; ABCB6, ATP-binding cassette sub-family B member 6; CPOX, coproporphyrinogen oxidase; PPOX, protoporphyrinogen oxidase; FECH, ferrochelatase; MFRN, mitoferrin; FLVCR, feline leukemia virus subgroup C receptor; HCP1/PCFT heme carrier protein 1/proton-coupled folate transporter; ABCG2, ATP-binding cassette sub-family G member 2; HO, heme oxygenase; Hx, Hemopexin.

During several pathological conditions, red blood cells undergo hemolysis and hemoglobin and heme are released into the circulation. Once released, free hemoglobin is captured by its carrier Hp and transported to the macrophages of the reticulo-endothelial system, where the complex is bound by the scavenger receptor CD163. When the buffering capacity of plasma Hp is exceeded, hemoglobin is quickly oxidized to methemoglobin, which releases free heme (Ascenzi et al., 2005). Ferriheme is then bound by Hx, by virtue of its high affinity (Figure 2). Hx is a 57-kDa acute phase plasma glycoprotein able to bind an equimolar amount of heme and to transport it into the circulation. Hx is expressed mainly in the liver, but also in the brain and retina (Tolosano et al., 1996, 2010). Hx is an acute phase response protein. The acute phase response is a complex systemic early-defence system activated by trauma, infection, stress, neoplasia, and inflammation. Most of these stimuli, in particular hemolytic stress and inflammatory stimuli, induce Hx synthesis (Tolosano and Altruda, 2002). Hx functions as a heme scavenger, maintaining lipophilic heme in a soluble state in aqueous environment and is essential in the re-utilization of heme-bound iron and prevention of heme-induced oxidative damage and cell death (Eskew et al., 1999). Hx acts as an antioxidant thanks to the ability to tightly bind heme, thus effectively reducing heme toxicity by 80–90% (Grinberg et al., 1999). Hx has the specific function to deliver heme to hepatocytes where the heme-Hx complex is internalized by receptor-mediated endocytosis. To date, the only known Hx receptor is the LDL receptor-related protein 1 (LRP 1), a multi-ligand scavenger receptor present on the surface of many cell types. Some studies have suggested that Hx can be recycled as an intact molecule to the extracellular milieu. However, Hvidberg et al. have shown that most Hx is degraded in lysosomes (Hvidberg et al., 2005). The binding of Hx to free heme limits the amounts of heme available as a catalyst of radical formation, makes the essential iron unavailable to invasive microorganisms and contributes to the recycling of iron, as heme iron enters the intracellular iron pool.

Once the scavenging capacity of plasma Hx is exhausted, free heme can still be scavenged by plasma albumin (~66 kDa) (Figure 2). Whether heme/albumin complexes are recognized by specific receptors allowing heme degradation via the HO system has not been established. Thus, the protective role of albumin against heme toxicity remains uncertain (Tolosano et al., 2010).

## Regulation of intracellular heme content

The control of intracellular heme content occurs at multiple levels. Here we focus on the regulation of heme synthesis, degradation and plasma membrane heme trafficking, which ensure the maintenance of appropriate intracellular heme concentration. Animal models discussed in this section are reported in Table 1.

**Table 1:** **Mouse models deficient for genes involved in the control of heme homeostasis**.

**Heme-related gene**	**Mouse model**	**Phenotype**	**References**
ALAS2	*Alas2*^−/−^ mice	Embryonic lethality; Impaired erythropoiesis; iron accumulation in primitive erythroid cells	Nakajima et al., 1999; Harigae et al., 2003
HEMOPEXIN (Hx)	*Hx*^−/−^ mice	Endothelial dysfunction, oxidative tissue damage, iron tissue iron redistribution following heme overload	Vinchi et al., 2008, 2013
HEME-OXYGENASE 1 (HO-1)	*Ho-*1^−/−^ mice	Hepatic and renal iron loading; splenic atrophy; macrophage depletion	Poss and Tonegawa, 1997; Kovtunovych et al., 2010
FLVCR1	*Flvcr1a/b*^−/−^ mice	Embryonic lethality; Impaired erythropoiesis; Reduced growth and skeletal malformations	Keel et al., 2008
	*Flvcr1a/b*^−/−^ postnatal mice	Macrocytic anemia	Keel et al., 2008
	*Flvcr1a*^−/−^ mice	Embryonic lethality; Hemorrhages, edema and skeletal malformations	Chiabrando et al., 2012
	Liver conditional *Flvcr1a*^−/−^ mice (*Flvcr1a*^*flox/flox*;^ *Alb-Cre*)	Hepatic heme and iron loading; reduced heme synthesis and cytochrome P450 expression/activity in hepatocytes	Vinchi et al., 2014
ABCG2	*Abcg2*^−/−^ mice	Photosensitization, PPIX accumulation	Jonker et al., 2002
HCP1	*Hcp1/PCFT*^−/−^ mice	Mortality at 3 months; systemic and cerebrospinal folate deficiency; severe macrocytic normochromic anemia; pancytopenia; liver iron accumulation	Salojin et al., 2011

### Control of heme synthesis

The first level of regulation of cellular heme content occurs at the level of heme synthesis control. Heme is synthesized through a series of eight enzymatic reactions (Figure 2). Work in the past decade has shown that heme synthesis is an almost ubiquitous process. The biosynthesis of heme starts in the mitochondrial matrix with the condensation of succinyl-CoA and glycine to form ALA, a reaction catalyzed by the enzyme ALAS. There are two isoforms of ALAS: *Alas1* gene is located on chromosome 3 in humans and codes for an ubiquitously expressed protein whereas *Alas2* gene is on the X chromosome and codes for an erythroid-specific protein (Bishop et al., 1990). The two isoforms of ALAS mainly differ for their mode of regulation, as discussed later in this section.

ALA is exported in the cytosol soon after its synthesis. The precise molecular mechanism by which ALA is transported through the two mitochondrial membranes is not completely understood. Two mitochondrial inner membrane proteins, SLC25A38 (solute carrier family 25, member 38) and the ATP-binding cassette transporter ABCB10 have been proposed to play this role.

Yeast lacking YDL119c, the ortholog of SLC25A38, shows a defect in the biosynthesis of ALA (Guernsey et al., 2009). Thus, it has been suggested that SLC25A38 could facilitate the production of ALA by importing glycine into mitochondria or by exchanging glycine for ALA across the mitochondrial inner membrane. Recently, Bayeva et al. reported that the silencing of ABCB10 causes a decrease of cellular and mitochondrial heme levels. The administration of ALA fully restores heme level in ABCB10-downregulated cells whereas Alas2 overexpression fails to do this. Thus, it has been proposed that ABCB10 could facilitate mitochondrial ALA synthesis or its export from mitochondria (Bayeva et al., 2013).

Both proteins are located on the inner mitochondrial membrane; the ALA transporter on the outer mitochondrial membrane still remains to be identified.

In the cytosol, two molecules of ALA are condensed to form the monopyrrole porphobilinogen, a reaction catalyzed by aminolevulinate dehydratase (ALAD). Then, the enzyme hydroxymethylbilane synthase (HMBS) catalyzes the head-to-tail synthesis of four porphobilinogen molecules to form the linear tetrapyrrole hydroxymethylbilane which is converted to uroporphyrinogen III by uroporphyrinogen synthase (UROS). The last cytoplasmic step, the synthesis of coproporphyrinogen III (CPgenIII), is catalyzed by uroporphyrinogen decarboxylase (UROD) (Ajioka et al., 2006). All the remaining steps of heme biosynthesis take place inside mitochondria, thus CPgenIII needs to be transported in the mitochondrial intermembrane space. It has been initially proposed that the ATP-binding cassette transporter ABCB6 could play this role (Krishnamurthy et al., 2006). However, data concerning the localization and function of ABCB6 in mitochondria are controversial. ABCB6 was also found to be expressed on the plasma membrane, in the Golgi compartment and in lysosomes. Some works even fail to detect ABCB6 in mitochondria (Paterson et al., 2007; Tsuchida et al., 2008; Kiss et al., 2012). In addition, ABCB6 has been associated to other functions unrelated to porphyrin homeostasis: ABCB6 contributes to anticancer drug resistance (Kelter et al., 2007); it was identified as the genetic basis of the Lan blood group antigen expressed on red blood cells (Helias et al., 2012); defects in *Abcb6* cause an inherited developmental defect of the eye, with no known relationship with porphyrins accumulation (Wang et al., 2012). Recently, it has been reported that Abcb6^−/−^ mice completely lack mitochondrial ATP-driven import of CPgenIII and shows the up-regulation of compensatory porphyrin and iron pathways. Abcb6^−/−^ mice are phenotypically normal; increased mortality and reduced heme synthesis were observed following phenylhydrazine administration thus suggesting that Abcb6 is essential during conditions of high porphyrin demand (Ulrich et al., 2012).

In the mitochondrial intermembrane space, CPgenIII is converted to protoporphyrinogen IX by the enzyme coproporphyrinogen III oxidase (CPOX), a homodimer weakly associated with the outside of the inner mitochondrial membrane. The following oxidation of protoporphyrinogen IX to protoporphyrin IX (PPIX) is catalyzed by protoporphyrinogen oxidase (PPOX), located on the outer surface of the inner mitochondrial membrane. Finally, ferrous iron is incorporated into PPIX to form heme in the mitochondrial matrix, a reaction catalyzed by the enzyme ferrochelatase (FECH) (Ajioka et al., 2006). It has been reported that FECH is part of a multi-enzyme complex composed by the mitochondrial iron importer MITOFERRIN1 (MFRN1) and the ATP-binding cassette transporter ABCB10. The association of FECH with MFRN1 allows the coupling of mitochondrial iron import and the integration of iron into PPIX ring while ABCB10 stabilizes MFRN1 expression (Chen et al., 2009, 2010).

Once synthesized, heme must be exported through the two mitochondrial membranes for incorporation into hemoproteins. The only mitochondrial heme exporter identified to date is the mitochondrial isoform of *Flvcr1* (Feline Leukemia Virus subgroup C Receptor 1) gene. Two different isoforms of FLVCR1 exist, FLVCR1a and FLVCR1b, expressed on the plasma membrane and mitochondria respectively. FLVCR1b derives from an alternative transcription start site located in the first intron of the *Flvcr1* gene thus resulting in the production of a shorter protein (Chiabrando et al., 2012). *Flvcr1a* transcript codes for a protein with 12 transmembrane domains (Tailor et al., 1999; Quigley et al., 2000) while *Flvcr1b* mRNA codes for a protein with just six transmembrane domains (Chiabrando et al., 2012). The role of FLVCR1b as a mitochondrial heme exporter is suggested by *in vitro* data indicating that its overexpression promotes heme synthesis whereas its silencing causes detrimental heme accumulation in mitochondria. Moreover, FLVCR1b expression is upregulated following the stimulation of heme synthesis *in vitro* (Chiabrando et al., 2012). According to its role as a mitochondrial heme exporter, FLVCR1b is essential for erythroid differentiation both *in vitro* and *in vivo* (as discussed in section Control of Heme Export). The submitochondrial localization of FLVCR1b is still unknown as well as its ability to interact with other mitochondrial transporters. Further work is needed to definitively understand how heme is transported across the two mitochondrial membranes.

The importance of controlling the rate of heme synthesis is highlighted by the fact that mutations in many genes coding for enzymes involved in this pathway cause specific pathological conditions characterized by the accumulation of toxic heme precursors (as discussed in section Pathological Conditions Associated with Alterations of Heme Synthesis). The regulation of heme synthesis mainly occurs at the level of ALAS, the first and rate-limiting enzyme of the heme biosynthetic pathway. It has been demonstrated that heme plays an essential role in the regulation of its own synthesis by regulating the expression of ALAS.

In non-erythroid cells, heme synthesis is dependent on the activity of ALAS1. It has been reported that ALAS1 is directly regulated by heme levels through several mechanisms: heme negatively controls the transcription (Yamamoto et al., 1982), translation (Sassa and Granick, 1970; Yamamoto et al., 1983) and stability (Hamilton et al., 1991) of *Alas1* mRNA. In addition, it has been shown that ALAS1 contains three heme regulatory motifs (HRMs). Heme binding to two of these HRMs, located in the mitochondrial targeting sequence of ALAS1, inhibits the transport of ALAS1 precursor protein in mitochondria (Yamauchi et al., 1980). The regulation of ALAS1 by heme represents a crucial negative feedback mechanism to maintain appropriate intracellular heme levels in non-erythroid cells, thus avoiding heme-induced oxidative damage.

In erythroid cells, heme synthesis is exclusively dependent on the activity of ALAS2. Contrary to ALAS1, heme does not inhibit the expression of ALAS2, as high amount of heme are required for the differentiation of erythroid progenitors. The expression of ALAS2 is controlled at multiple levels. The transcription of *Alas2* is regulated by erythroid-specific transcription factors, like GATA1 (Surinya et al., 1998; Kaneko et al., 2013). At the post-transcriptional level, the expression of ALAS2 is regulated by iron availability. The 5' untranslated region of *Alas2* mRNA contains an iron responsive element (IRE) which interacts with iron regulatory protein (IRP) 1 and 2. The IRE binding activity of IRPs is regulated by cellular iron. In iron-deficient cells, the binding of IRPs to the 5'IRE inhibits the translation of *Alas2* mRNA. In iron-repleted cells, IRPs are degraded thus stimulating the translation of *Alas2* mRNA. This mechanism ensures the coordination of heme synthesis to the availability of iron thus avoiding the production of potentially toxic heme precursors when iron concentrations are limiting. The regulation of the IRE-binding activity of IRPs by cellular iron occurs through several mechanisms. The IRE binding activity of IRP1 is controlled by iron-sulfur clusters assembly while that of IRP2 by its oxidation, ubiquitination and degradation by the proteasome (Hentze et al., 2010). This latter process is dependent on iron but also on heme availability. It has been reported that IRP2 contains a HRM; heme binding to the HRM mediates the oxidation of IRP2 that triggers its ubiquitination and degradation (Yamanaka et al., 2003; Ishikawa et al., 2005). Thus, during the differentiation of erythroid progenitors, increased cellular iron level stimulates the translation of *Alas2* mRNA by inducing the degradation of IRPs. The following accumulation of heme contributes to the oxidation and degradation of IRP2, further enhancing heme synthesis. This positive feedback mechanism allows a sustained production of heme for hemoglobin synthesis in differentiating erythroid progenitors.

### Control of heme incorporation into hemoproteins

The rate of *de novo* heme synthesis has to be proportionate to its rate of incorporation into newly synthesized apo-hemoproteins. This is obtained at different levels via the control of heme synthesis as well as apo-hemoproteins synthesis. As reported in section Heme as a Modulator of Gene Expression and Cell Proliferation/Differentiation, heme itself can induce the transcription and the expression of several apo-hemoproteins, such as hemoglobin, myoglobin, and neuroglobin (Bruns and London, 1965; Tahara et al., 1978; Zhu et al., 1999, 2002; Correia et al., 2011), as well as cytochromes and many other heme-containing proteins. This evolutionary conserved strategy prevents intracellular heme accumulation, presumably limiting heme cytotoxicity (Figure 2). Additionally, in non-erythroid cells, heme is able to regulate its own synthesis and degradation via inhibition of ALAS1 expression/activity and induction of HO-1 expression/activity, thus properly balancing the amount of synthesized heme with that one incorporated into hemoproteins or catabolized (Zheng et al., 2008; Correia et al., 2011).

### Control of heme degradation

HO is the primary enzyme involved in heme degradation and plays an important role in the protection of cells from heme-induced oxidative stress. It is a 32 kDa protein, mainly located on membranes of the smooth endoplasmic reticulum, able to break down the pro-oxidant heme into the antioxidant biliverdin, the vasodilator carbon monoxide (CO) and iron (Fe^2+^) (Figure 2) (Gozzelino et al., 2010; Tolosano et al., 2010). Biliverdin is then reduced to bilirubin by the enzyme biliverdin reductase. To date, three isoforms of HO have been identified: HO-1, HO-2, and HO-3.

HO-1 is highly inducible by a variety of stimuli including oxidative stress, heat shock, hypoxia, ischemia-reperfusion, lipopolysaccharide, heavy metals, cytokines and its substrate heme. Heme is the most potent physiologic inducer of HO-1. The enzyme activity was shown to increase in many tissues, including the liver, kidney, adrenals, ovaries, lung, skin, intestine, heart, and peritoneal macrophages. HO-2 is ubiquitously expressed and participates in the normal heme capturing and metabolism. The isoenzymes HO-1 and HO-2 are products of two different genes. The two forms show only 45% aminoacid homology but they share a region with 100% secondary structure homology, that corresponds to the catalytic site (Maines, 1988). HO-3 has poor heme-degrading capacity (Wagener et al., 2003).

Herein, we focus on the inducible isoform HO-1.

HO-1 plays a vital function in heme degradation and protects against heme-mediated oxidative injury. Overexpression of HO-1 is associated to the resolution of inflammation through the generation of beneficial molecules like CO, bilirubin, and ferritin resulting from catabolism of toxic heme (Wagener et al., 2001, 2003; Kapturczak et al., 2004). Bilirubin efficiently scavenges peroxyl radicals, thereby inhibiting lipid peroxidation, attenuating heme-induced oxidative stress, cell activation and death (Dore et al., 1999; Soares et al., 2004; Kawamura et al., 2005). CO controls the activity of several heme proteins and causes vasodilation. It also exerts anti-inflammatory effects by inhibiting the expression of pro-inflammatory cytokines (Ndisang et al., 2003; Beckman et al., 2009). Finally, ferritin, by sequestering toxic free iron, limits microrganism growth and ROS production.

HO-1 confers cytoprotection against different forms of programmed cell death, including apoptosis driven by heme and tumor necrosis factor (Gozzelino and Soares, 2011). This cytoprotective effect is driven by heme degradation *per se* as well as by its end products, CO and biliverdin/bilirubin (Yamashita et al., 2004; Gozzelino et al., 2010). CO can exert cytoprotective effects via the modulation of cellular signal pathways, including the p38 mitogen activating protein kinase (Ndisang et al., 2003). In addition, CO can bind Fe in the heme pockets of hemoproteins, inhibiting heme release and preventing its cytotoxic effects (Seixas et al., 2009; Ferreira et al., 2011). On the other hand, biliverdin has been described to participate in an antioxidant redox cycle in which, once produced by HO, biliverdin is reduced to bilirubin by biliverdin reductase. This is followed by the subsequent oxidation of bilirubin by ROS back to biliverdin, forming a catalytic antioxidant cycle that is driven by NADPH, the reducing cofactor of biliverdin reductase. This cycle has the ability to strongly suppress the oxidizing and toxic potential of hydrogen peroxide and other ROS, thus acting as one of the most powerful anti-oxidant physiological system.

Another detoxifying system is represented by ferritin, an evolutionarily conserved Fe sequestering protein that acts as the major intracellular depot of non-metabolic iron (Balla et al., 1992a,b; Berberat et al., 2003; Cozzi et al., 2004; Pham et al., 2004). Ferritin is a multimeric protein composed of 24 subunits of two types, the heavy chain (H-Ft) and the light chain (L-Ft) and has a very high capacity for storing iron (up to 4500 mol of iron per mol of ferritin). H-Ft manifests ferroxidase activity that catalyses the oxidation of ferrous iron to ferric iron, thus favoring its storage in L-Ft (Hentze et al., 2004) and limiting its participation in the production of free radicals via Fenton reaction (Pham et al., 2004). Together, HO and ferritin allow rapid shifting of iron from heme into ferritin core where it is less available to catalyze deleterious reactions. By increasing the expression of HO-1 and ferritin, cells can survive to lethal heme-induced oxidative stress (Balla et al., 2005; Gozzelino and Soares, 2013).

### Control of heme export

Recent evidence demonstrated that also heme export out of the cell significantly contributes to the regulation of intracellular heme levels. Two heme exporters located at the plasma membrane have been identified to date: FLVCR1a and ABCG2 (ATP-Binding Cassette, subfamily G, member 2) (Figure 2).

#### The plasma heme exporter FLVCR1a

FLVCR1a was originally identified and cloned as a cell-surface protein receptor for feline leukemia virus subgroup C, causing pure red blood cell aplasia in cats (Tailor et al., 1999; Quigley et al., 2000). The role of FLVCR1a as a plasma membrane heme exporter is suggested by several *in vitro* data. Indeed, the overexpression of FLVCR1a in NRK or HeLa cells causes a slight but significant decrease of heme content whereas its silencing in FEA or HeLa cells enhances heme level (Quigley et al., 2004; Chiabrando et al., 2012). In addition, the ability of FLVCR1a to export cytoplasmic heme has been demonstrated using zinc mesoporphyrin (ZnMP), a fluorescent heme analog, and ^55^Fe-heme (Quigley et al., 2004). The heme export activity of FLVCR1a is regulated by the presence of plasma proteins with high affinity for heme, like Hx (Yang et al., 2010).

**Figure 3:**
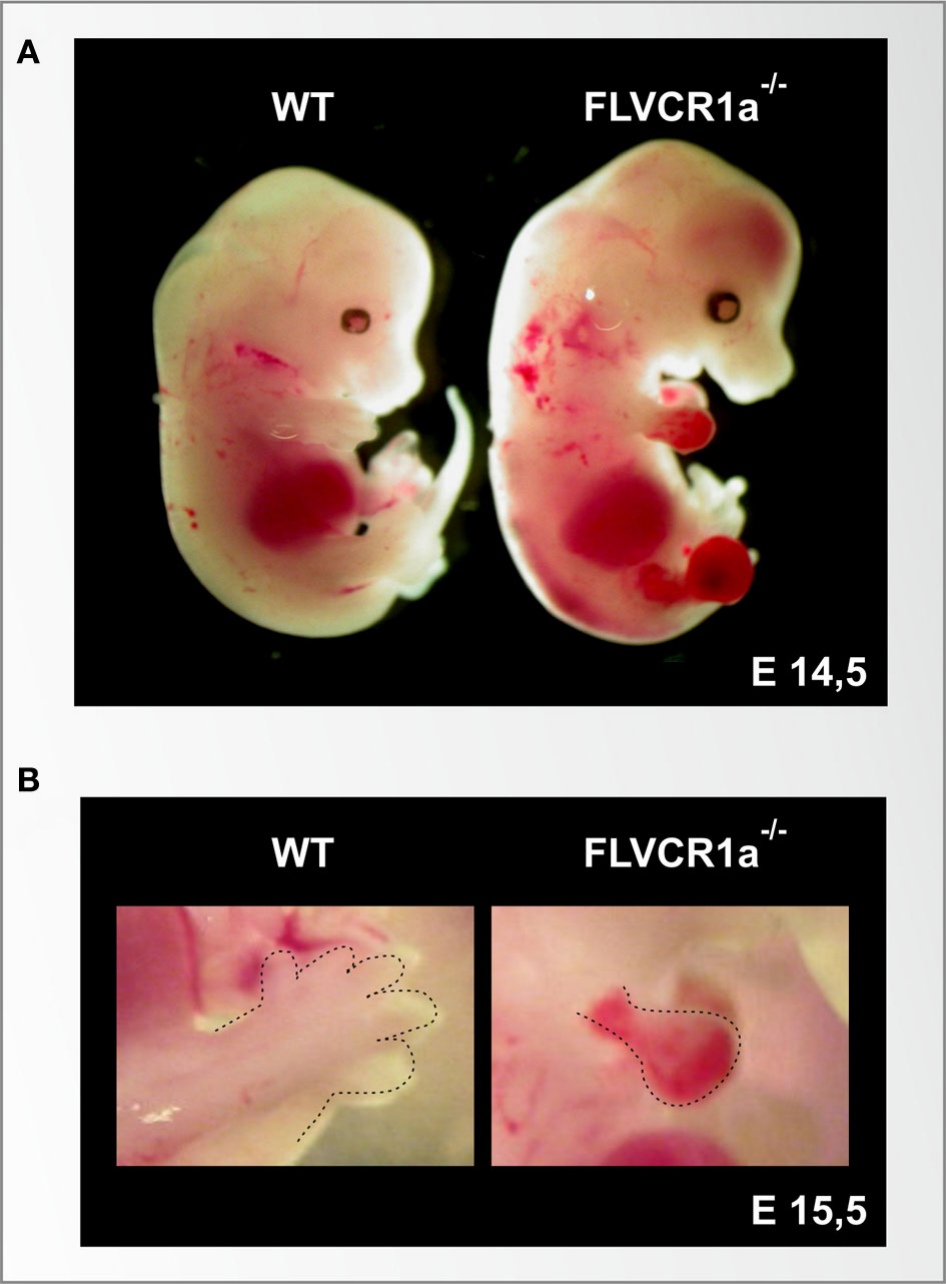
**The loss of the heme exporter FLVCR1a in mice causes embryonic lethality, skeletal malformation and extended hemorrhages. (A)** Stereoscopic view of a wild-type (left) and a *Flvcr1a*^−/−^ (right) embryo at the embryonic stage of E14,5. At this stage, the wild-type embryo shows normal skeletal structure, with fully formed limbs. The *Flvcr1a*^−/−^ embryo shows extended hemorrhages and edema through the body, in particular in the limbs, back and head. *Flvcr1a*^−/−^ embryos show skeletal malformations, as suggested by the absence of the lower jaw and properly formed digits. **(B)** An enlarged view of E15,5 wild-type and *Flvcr1a*^−/−^ anterior limbs (marked with a broken line). In the *Flvcr1a*^−/−^ embryo, the limbs show severe hemorrhage, leading to impairment in limb and toe formation.

**Figure 4:**
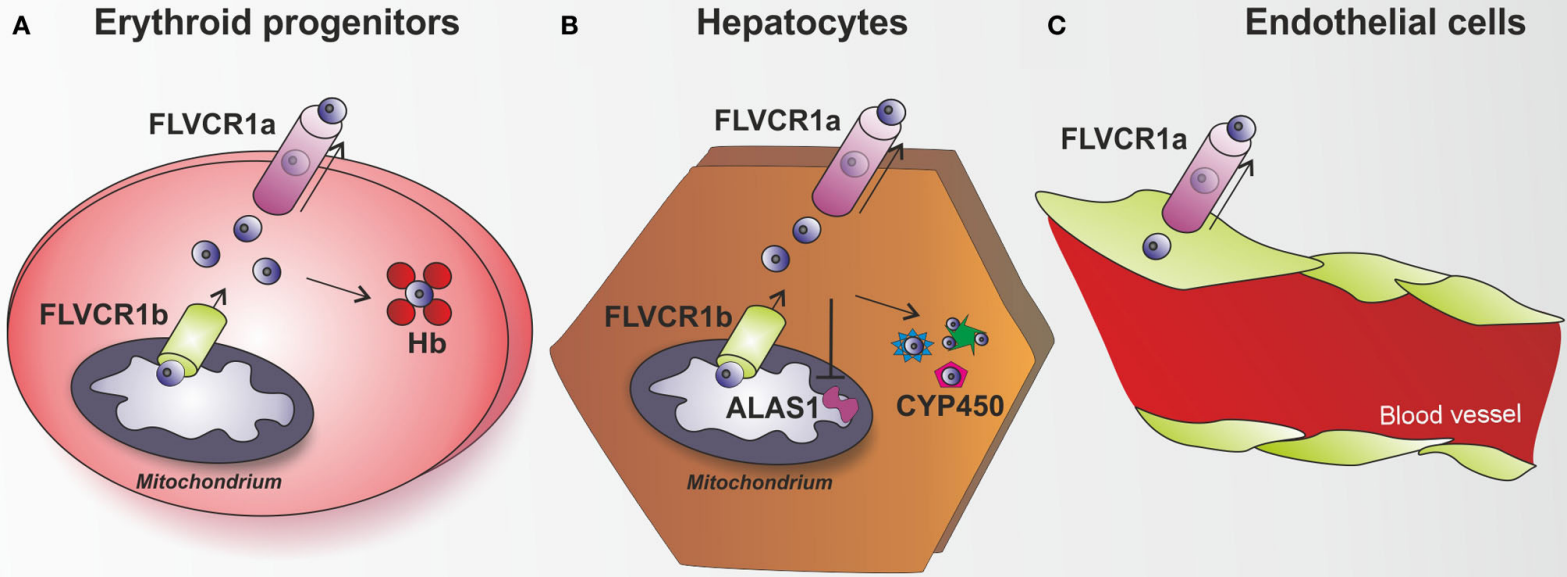
**The heme exporter FLVCR1a acts as a new heme detoxifying system. (A)** Erythroid progenitors are able to synthesize and handle high amount of heme, in view of their hemoglobin (Hb)-mediated oxygen transport activity. FLVCR1b acts as a mitochondrial heme exporter to allow newly formed heme release from the mitochondrion to the cytosol, where it is incorporated into hemoproteins. FLVCR1a has been described as a system involved in the control of heme levels inside erythroid progenitors. By mediating heme export out of these cells, FLVCR1a regulates intracellular heme amount, thus limiting free heme toxicity and oxidative damage. **(B)** Hepatocytes have the highest rate of heme synthesis after the erythroid progenitors. Hepatic heme is mostly used for synthesis of P450 enzymes, which metabolize endogenous compounds and xenobiotics. FLVCR1a mediates heme export out of hepatocytes, thus maintaining hepatic heme homeostasis and controlling cell oxidative status. FLVCR1a export function allows the maintenance of a proper cytosolic heme pool that matches cell need for new hemoprotein generation (e.g., cytochrome P450). Block of heme export causes heme pool expansion leading to the inhibition of heme synthesis and the reduction of cytochrome activity. **(C)** A similar role for FLVCR1a was proposed to occur in endothelial cells. *Flvcr1a*^−/−^ embryos show reduced vascular arborization, potentially due to altered endothelial integrity, suggesting that the lack of FLVCR1a leads to intracellular heme overload and oxidative stress. Endothelial cells are highly sensitive to heme overload and, in this context, FLVCR1a function could be of crucial importance to export heme excess, thus maintaining heme homeostasis and controlling heme-induced oxidative stress.

The analysis of the role of FLVCR1a *in vivo* is limited by the complexity of the available mouse models of *Flvcr1* deficiency (Table 1). It was initially reported that FLVCR1a plays an essential role during erythropoiesis, by preventing the toxic accumulation of heme in erythroblasts (Keel et al., 2008). This hypothesis was suggested by the observation that mice lacking *Flvcr1* die *in utero* due to an impairment of erythroid differentiation at the proerythroblast stage. Similarly, post-natal mice lacking *Flvcr1* show a block of erythroid maturation leading to hyperchromic, macrocytic anemia and reticulocytopenia (Keel et al., 2008). Following the identification of FLVCR1b, it was realized that these mouse models were likely lacking both FLVCR1a and FLVCR1b, as they were generated by the deletion of the third exon of the *Flvcr1* gene, which is common to both *Flvcr1* isoforms. Thus, the described phenotype was due to FLVCR1a and/or FLVCR1b deficiency. The specific expression of the two FLVCR1 isoforms in these mouse models still remain to be experimentally verified. Recently, the generation and analysis of *Flvcr1a*^−/−^ mice suggested that the previously described phenotype was mainly due to the absence of FLVCR1b, since mice lacking FLVCR1a but still expressing FLVCR1b show normal erythroid differentiation and die *in utero* due to severe hemorrhages, edema and skeletal malformations (Figure 3). Taken together, these data suggest that FLVCR1b, by exporting heme from mitochondria, is essential for fetal erythroid differentiation (Chiabrando et al., 2012). These data do not exclude a role for FLVCR1a during erythropoiesis; probably the two FLVCR1 isoforms cooperate to determine the appropriate heme level needed for erythroid differentiation (Figure 4A).

As FLVCR1a is ubiquitously expressed, it has been hypothesized that its heme export activity could be relevant in different tissues. Post-natal mice lacking *Flvcr1* show iron overload in hepatocytes, duodenal enterocytes and splenic macrophages (Keel et al., 2008). In this mouse model the observed phenotype could not be easily attributed to one of the two isoforms since both FLVCR1a and FLVCR1b were deleted. The specific contribution of FLVCR1a isoform to heme export became evident in conditional mice lacking FLVCR1a expression in hepatocytes. These mice accumulate heme and iron in the liver as a consequence of FLVCR1a suppression (Vinchi et al., 2014). Additionally, they show HO and ferritin upregulation, together with alteration of the hepatic oxidative status and induction of the antioxidant genes. These data suggest that heme is normally exported intact from these cells and that in the absence of FLVCR1a, the heme degrading and iron storage systems are upregulated as an attempt to compensate for the lack of heme export (Figure 4B).

The analysis of conditional *Flvcr1a*^−/−^ mice in hepatocytes demonstrated a crucial role for FLVCR1a in the maintenance of hepatic heme homeostasis and hemoprotein function. Interestingly, FLVCR1a expression was found upregulated during cytochrome induction, suggesting that hepatic heme export activity of FLVCR1a was closely associated with heme biosynthesis required to sustain new cytochrome synthesis. Indeed, the lack of FLVCR1a in hepatocytes caused the expansion of the cytosolic heme pool that was responsible for the early inhibition of heme synthesis and increased degradation of heme. As a result, the expression as well as the activity of cytochromes P450 was reduced. These findings indicate that FLVCR1a-mediated heme export is crucial to control intracellular heme levels that in turn regulate heme synthesis, thus determining cytochrome function in the liver (Vinchi et al., 2014).

Additionally, the analysis of *Flvcr1a*^−/−^ embryos highlighted a previously unrecognized role for FLVCR1a in the maintenance of endothelial integrity. *Flvcr1a*^−/−^ embryos are characterized by reduced vasculature development and complexity. This is particularly evident in the limbs and tail, where vessels do not form properly and branching is severely compromised (Chiabrando et al., 2012) (Figure 3). The molecular mechanism leading to the observed phenotype is still unknown. Interestingly, FLVCR1a is regulated at the transcriptional level by hypoxia, which has a well-established role in angiogenesis and vasculogenesis (Fiorito et al., 2014). We hypothesize a role for FLVCR1a in preventing heme-induced oxidative stress in endothelial cells (Figure 4C). FLVCR1a mediated heme export could work in strong association with HO-1 to determine the appropriate amount of heme in endothelial cells. It is well established that HO-1 plays a pivotal role in the regulation of vascular biology (Belcher et al., 2006, 2010; Stocker and Perrella, 2006; Kim et al., 2011). For this reason, it will be interesting to investigate the role of FLVCR1a in hemolytic disorders characterized by enhanced heme-induced oxidative stress in endothelial cells.

The understanding of FLVCR1a subcellular localization, particularly in polarized cell types, will help to gain new insight in FLVCR1a role and function. To date, it has been reported that FLVCR1a localizes on the sinusoidal membrane of hepatocytes, likely exporting heme in the bloodstream (Vinchi et al., 2014). These results have been obtained by studies on the overexpressed protein and are limited by the lack of specific antibodies for this exporter that prevents any analysis of the localization of endogenous FLVCR1a. Future work is required to specifically address FLVCR1a localization in different cell types *in vivo*.

#### The plasma heme exporter ABCG2

ABCG2 (ATP-binding cassette, sub-family G, member 2; also known as BRCP, breast cancer resistance protein) is a member of the ABC transporter family that was originally found to confer drug resistance in breast cancer cells (Doyle et al., 1998). The *Abcg2* gene is located on human chromosome 4q22 and it consists of 16 exons and 15 introns (Bailey-Dell et al., 2001). ABCG2 is a “half transporter” as it has only one ABC cassette in a single polypeptide chain of 70 kDa that consists of six transmembrane domains. Only the homodimer is functional (Kage et al., 2002). *Abcg2* expression is regulated by hypoxia through Hypoxia Inducible Factor (HIF) 1 (Krishnamurthy et al., 2004). ABCG2 is localized at the plasma membrane and it is expressed in several tissues including hepatic canalicular membranes, renal proximal tubules, intestinal epithelium and placenta (Doyle and Ross, 2003). In all these tissues, ABCG2 detoxifies drugs, toxins and metabolites (Bailey-Dell et al., 2001). Moreover, ABCG2 is expressed in a sub-population of hematopoietic stem cells. It prevents cytotoxicity of chemotherapics and confers resistance to hypoxic conditions (Krishnamurthy et al., 2004).

ABCG2 has a wide substrate specificity (Keppler and Konig, 2000). Recently, genetic studies in human disease established that ABCG2 functions as a urate transporter that promotes urate excretion in the kidney as discussed in section Hyperuricaemia and Gout (Qiu et al., 2006). The role of ABCG2 as a heme transporter was serendipitously discovered when *Abcg2*^−/−^ mice fed a modified diet developed skin photosensitivity (Jonker et al., 2002). This was caused by the accumulation of pheophorbide, a degradation product of chlorophyll present in the diet, structurally similar to PPIX. *Abcg2*^−/−^ mice were found to accumulate PPIX and other porphyrin-like compounds in erythrocytes and other cells implying that ABCG2 has a role in cellular efflux of these compounds (Jonker et al., 2002). In addition, it has been demonstrated that ABCG2 is precipitated by hemin-agarose and it exports the heme analog ZnMP in K562 cells transfected with the human *Abcg2* cDNA (Krishnamurthy et al., 2004; Desuzinges-Mandon et al., 2010). Nevertheless, the physiologic porphyrin substrate of ABCG2 seems to be PPIX as erythroid progenitors of *Abcg2*^−/−^ mice accumulate this compound and erythroid cells overexpressing ABCG2 have reduced levels of PPIX (Krishnamurthy and Schuetz, 2005; Zhou et al., 2005). Interestingly, erythroid progenitors of *Abcg2*^−/−^ mice are more sensitive to hypoxic conditions. Since *Abcg2* expression is induced by HIF1, ABCG2 is thought to confer a strong survival advantage to stem cells under hypoxic stress by reducing intracellular porphyrin content (Krishnamurthy et al., 2004).

Despite of porphyrin accumulation in erythroid progenitors, *Abcg2*^−/−^ mice do not show porphyria or an overt anemic phenotype. This might indicate that pophyrin export does not significantly contribute to intracelluar porphyrin homeostasis and/or that compensatory mechanisms are activated when *Abcg2* is lacking. Alternatively, this could also indicate that ABCG2 is not a physiologic porphyrin exporter. Nevertheless, ABCG2 expression is induced in HeLa cells after the stimulation of heme synthesis (Chiabrando et al., 2012), supporting the conclusion that, in some way, it is involved in this process.

Based on its localization at the apical membrane of duodenal enterocytes, it has been hypothesized that ABCG2 could transport excess heme or porphyrin from the enterocyte to the lumen. Similarly, it could be involved in porphyrin/heme detoxification through the biliary system and/or the kidney (Latunde-Dada et al., 2006; Krishnamurthy et al., 2007). Consistently, ABCG2 was found to cooperate with HO-1 to protect kidney cells against heme-induced cytotoxicity (Wagener et al., 2013). Finally, *Abcg2* deficiency was found to increase oxidative stress and to exacerbate cognitive/memory deficit in a mouse model of Alzheimer's disease (Zeng et al., 2012).

Thus, ABCG2 is a plasma membrane transporter for a wide variety of substrates including urate porphyrin/heme, chemiotherapeutics, antibiotics, xenobiotics, and food metabolites. If, other than in urate transport and in drug metabolism, ABCG2 plays a role in heme export under physiologic or pathologic conditions remains to be elucidated.

### Control of heme import

A further level of control of intracellular heme content is represented by the modulation of heme import inside the cells (Figure 2). To date the only known proteins with a well-established function as heme importers are HRGs (Rajagopal et al., 2008; White et al., 2013), reviewed by Hamza et al. in the present Research Topic. Two other putative heme importers have been described, the Feline leukemia virus subgroup C receptor 2 (FLVCR2) and the Heme carrier protein 1/Proton-coupled folate transporter (HCP1/PCFT).

#### The heme importer FLVCR2

FLVCR2 is the second member of the SLC49 family of heme transporters, a family also including the founding member FLVCR1 (SLC49A1), and other two proteins, MFSD7 (SLC49A3) and DIRC2 (SLC49A4) (Khan and Quigley, 2013). The SLC49 family belongs to the Major Falicitator Superfamily (MFS) of secondary active permeases that transports small solutes across membranes in response to chemico-osmotic gradients, contributing to the maintenance of normal cell homeostasis (Pao et al., 1998).

The gene encoding FLVCR2 contains 10 exons, is located on chromosome 12D2 in mouse and 14q24 in human (Quigley et al., 2000) and is highly homologous to the *Flvcr1* gene. FLVCR2 protein shares about 60% amino acid sequence identity with FLVCR1. A variant transcript exists, encoding a shorter hypothetical protein that differs in the N-terminal and whose significance is unknown (Brown et al., 2006).

*Flvcr2* mRNA is found ubiquitously, with the highest transcript levels observed in human brain, placenta, lung, liver, kidney and hematopoietic tissues (Duffy et al., 2010). In mouse, its expression has been reported in brain, spinal cord (Lein et al., 2007) and in the columnar cells overlying the fetal blood vessels in the placental yolk sac at day E20 (Brasier et al., 2004).

The FLVCR2 protein is composed by 12 predicted transmembrane domains and six presumptive extracellular loops, it is not N-linked glycosylated and shows a molecular mass of about 55-kDa, while its truncated variant weights about 40 kDa (Brown et al., 2006). Unfortunately, to date a FLVCR2-specific antibody is not available, so protein expression in the different tissues and cell compartments has not been determined. Nevertheless, the FLVCR2 functions, described below, strongly indicate its localization on the cell plasma membrane.

Brasier et al. (2004) proposed a putative role for FLVCR2 as a calcium transporter, based on its expression in murine and human tissues characterized by rapid calcium exchange. Although this function has not been formally excluded by subsequent studies, alternative roles for FLVCR2 have emerged. FLVCR2 has been identified as the receptor for the FY981 variant of FeLV-C retrovirus, a variant that can also use FLVCR1 and the thiamine transporter 1 to infect cells (Shalev et al., 2009). Unlike FLVCR1, FLVCR2 was unable to export heme (Quigley et al., 2004). Conversely, FLVCR2 was postulated to be a heme importer since (i) the overexpressed protein coprecipitated with hemin-agarose, (ii) human cells overexpressing FLVCR2 showed an enhanced uptake of ZnMP, and (iii) *Xenopus* oocytes expressing human FLVCR2 showed increased uptake of [55Fe]hemin (Duffy et al., 2010). Nevertheless, it has to be noted that the binding of FLVCR2 to hemin-agarose was not very efficiently competed by free hemin and the transport assays showed only a two-fold increase of ZnMP or ^55^Fe-heme uptake when FLVCR2 was overexpressed. Furthermore, studies in yeast failed to demonstrate a heme transport function for FLVCR2 (Yuan et al., 2012). Finally, genetic studies in humans associated *Flvcr2* mutation to the Fowler syndrome, a disorder with no obvious link to heme metabolism (see section Fowler Syndrome). Thus, the assumption that FLVCR2 is a heme importer is not definitive and further studies are needed to fully address the substrate specificity of this transporter.

#### The heme importer HCP1/PCFT

HCP1/PCFT belongs to the SLC46 family of the MFS. The gene encoding HCP1/PCFT spans five exons and four introns and is located on chromosome 11B5 in mouse and 17q11 in human. The HCP1/PCFT protein is highly conserved among different species, sharing about 90% similarity between human and mouse or rat (Qiu et al., 2006). Furthermore, it shows a significant homology to bacterial metal tetracycline transporters, likely due to similarities in the structures of the substrates specific for HCP1/PCFT and this kind of transporters (Shayeghi et al., 2005).

*Hcp1/Pcft* transcript is abundant in the mouse duodenum after weaning and in the adult rat liver (Shayeghi et al., 2005), but a weak expression can also be detected in the mouse kidney (Shayeghi et al., 2005) and in many other mouse tissues (Salojin et al., 2011). No expression was observed in mouse placenta and ileum (Shayeghi et al., 2005) and in mouse adipose tissue or total bone tissue including bone marrow (Salojin et al., 2011). In human tissues, two different *Hcp1/Pcft* transcripts were observed, the shorter one being dominant. Both transcripts were detected in kidney, liver, placenta, duodenum and spleen, and to a lesser extent in jejunum, ileum, cecum, colon, rectum, and testis. Very low *Hcp1/Pcft* mRNA levels were observed in human brain, lung, stomach, heart and muscle (Qiu et al., 2006). Furthermore, the *Hcp1/Pcft* transcript was found in human macrophages (Schaer et al., 2008), in retina and in retinal pigment epithelium (Sharma et al., 2007). Finally, *Hcp1/Pcft* mRNA was also observed in several cell lines, like hepatoma, neuroblastoma and macrophage cell line (Shayeghi et al., 2005), in primary rat astrocytes (Dang et al., 2010) and in the Caco2 human adenocarcinoma cell line (Qiu et al., 2006).

Duodenal *Hcp1/Pcft* mRNA levels are not influenced by systemic iron amount or by increased ineffective erythropoiesis and IREs were not observed in the 5' and 3' untranslated regions of *Hcp1/Pcft* transcript (Shayeghi et al., 2005). Conversely, *Hcp1/Pcft* mRNA levels strongly increase upon hypoxia, despite the absence of hypoxia responsive elements in the promoter sequence of the *Hcp1/Pcft* gene. Moreover, the anti-inflammatory agents glucocorticoids increase *Hcp1/Pcft* mRNA in macrophages (Schaer et al., 2008), while the lipopolysaccharide and Toll-like receptors agonists lead to suppression of *Hcp1/Pcft* transcript in these cells.

The HCP1/PCFT protein contains 459 amino acids, shows a molecular mass of about 50 kDa, is composed of 9 (Shayeghi et al., 2005) or 12 (Sharma et al., 2007) predicted transmembrane domains and is localized both in the plasma membrane (particularly the apical membrane of polarized cells like enterocytes) and in subcellular vesicles (Shayeghi et al., 2005; Yanatori et al., 2010). In non-polarized cells, it could also localize in the lysosome (Yanatori et al., 2010), and in human macrophages it has been detected in the endosome (Schaer et al., 2008). The localization of HCP1/PCFT is highly influenced by changes in cellular iron stores, accumulating at the brush border membrane in iron-deficiency, and in the cytoplasm in iron-loaded conditions (Shayeghi et al., 2005).

HCP1/PCFT was initially identified as a heme importer (Shayeghi et al., 2005). Nevertheless, Qiu et al. (2006) reported that HCP1/PCFT is a folate transporter (Qiu et al., 2006). Considering that the K_*m*_ at pH 6.5 for 5-methyltetrahydrofolate was measured at 0.8 μM whereas the K_*m*_ at neutral pH for hemin was 125°μM, it appears that HCP1/PCFT is a poor heme transporter. This conclusion is strengthened by the finding that the gene coding for HCP1/PCFT was found mutated in patients with hereditary folate malabsorption (Qiu et al., 2006) (see section Hereditary Folate Malabsorption Syndrome).

Here, we report some other considerations on the putative role of HCP1/PCFT as a heme importer that could be relevant only in some cell types or in particular physiologic or pathologic situations. The expression of HCP1/PCFT in the duodenum and liver indicate that this protein might be important both for the import of dietary heme from the gut lumen to the enterocytes and for the uptake of plasma heme by non-intestinal tissues (Shayeghi et al., 2005). Moreover, it has been proposed that it could be involved in the export of hemoglobin-derived heme from the endosome to the cytoplasm of macrophages (Schaer et al., 2008). In addition to free heme import, both heme-arginine and heme-bovine serum albumin are able to donate heme for uptake via HCP1/PCFT. Nevertheless, in primary rat astrocyte cultures the greatest amount of heme accumulation was measured in the absence of Hx/albumin-containing fetal calf serum (Dang et al., 2010). The transport of heme by HCP1/PCFT is a saturable and energy/temperature-dependent process. HCP1/PCFT is able to import other substrates containing a porphyrin ring, as ZnMP and protoporphyrin (Shayeghi et al., 2005; Qiu et al., 2006).

The energy source for HCP1/PCFT-mediated heme transport is unknown, as no adenosine triphosphate binding motifs were observed in its sequence. Nevertheless, the prevalent hypothesis is that, as for folates uptake, it could utilize the co-transport of protons along a favorable concentration gradient to drive the transport of heme (Shayeghi et al., 2005).

## Pathological conditions associated with alterations of heme metabolism

### Pathological conditions associated with alterations of extracellular heme scavenging

Many pathological conditions are associated with hemolysis and extracellular heme release. Among them, sickle cell anemia, β-thalassemia, malaria, paroxysomal nocturnal hemoglobinuria are the most paradigmatic (Gozzelino et al., 2010). In all these conditions, the pool of circulating Hx is diminished and, consequently, the plasma heme scavenging capacity is strongly reduced.

In the last decade, the use of a knockout mouse model for Hx was valuable to elucidate its function as well as its potential use as a therapeutic molecule in the prevention of heme adverse effects (Table 1). Additionally, other functions of Hx not clearly related to its role as an heme scavenger, have also been described thanks to the use of knock-out mice (Fagoonee et al., 2008; Spiller et al., 2011; Rolla et al., 2013).

Works on Hx^−/−^ mice have demonstrated that these animals have alterations in heme/iron homeostasis in the duodenum (Fiorito et al., 2013) and brain (Morello et al., 2009, 2011), are highly sensitive to acute hemolysis (Vinchi et al., 2008) and show a defective recovery after intravascular hemolysis, suffering from severe renal damage associated with iron loading and lipid peroxidation (Tolosano et al., 1999, 2002). After acute heme overload, Hx^−/−^ mice develop severe hepatic red blood cell congestion associated to cell iron redistribution, lipid peroxidation and inflammation. This is likely due to the increased endothelial activation and vascular permeability occurring in Hx^−/−^ mice compared to wild-type controls (Vinchi et al., 2008). Endothelial activation is a proinflammatory and procoagulant state of the endothelial cells lining the lumen of blood vessels. This state is mainly characterized by an increased expression of adhesion molecules on endothelial cell surface, which promotes the adherence of leukocytes as well as platelets and red blood cells, thus favoring inflammation, clot and eventually thrombus formation. According to its function as a free heme scavenger, Hx is expected to counteract heme toxicity on the vascular endothelium and increasing experimental evidence is supporting this concept.

Similarly, sickle cell disease and β-thalassemia mice show heme-driven endothelial activation, vaso-occlusion and cardiovascular dysfunction that could be efficiently recovered through the administration of an Hx-based therapy (Vinchi et al., 2013). This recent observation further strengthens the concept that heme triggers vascular inflammation and damage, and emphasizes the importance of Hx in counteracting heme-driven cardiovascular dysfunction associated with hemolytic conditions. This could have relevance, in the future, for therapeutic interventions against cardiovascular and endothelial dysfunctions in hemolytic patients (Vinchi and Tolosano, 2013).

Malaria is another well-known hemolytic condition, associated with the accumulation of high concentrations of free heme in plasma. The appearance of hemoglobin and heme in plasma has been linked to the development of cerebral malaria, which remains the most severe and difficult to treat complication of the infection (Ferreira et al., 2008; Seixas et al., 2009). The potential protective effect of both Hp and Hx in this pathology still needs to be elucidated.

Recently, hemolysis has been observed to occur after red blood cell transfusion, one of the most common therapeutic interventions in medicine. Over the last two decades, however, transfusion practices have been restricted, limiting unnecessary transfusions (Barr and Bailie, 2011). The adverse effects of transfusions seem to be mainly related to the storage period between blood donation and transfusion (Wang et al., 2012). Transfusions of old blood in animal models result in both intravascular and extravascular hemolysis and cause hypertension, acute renal failure, hemoglobinuria, and vascular injury. Conversely, old blood transfusion together with the hemoglobin scavenger Hp attenuated most of the transfusion-related adverse effects (Baek et al., 2012; Schaer et al., 2013b). Whether a similar protection, alone or in association with Hp, could be afforded by Hx still needs to be explored.

Not long ago, severe sepsis was found accompanied by hemolysis and Hx exhaustion in mice (Larsen et al., 2012). Larsen and coworkers showed that the administration of exogenous Hx was protective against organ injury and prevents the lethal outcome of severe sepsis in mice (Larsen et al., 2010). The protective effect in this model is related to the ability of Hx to counteract heme proinflammatory effects upon pathogen infection.

Furthermore, a neuroprotective effect for Hx was also described. Hx was found expressed by cortical neurons and present in mouse cerebellum, cortex, hippocampus, and striatum. Upon experimental ischemia, neurologic deficits as well as infarct volumes in the brain were increased in Hx deficient mice, indicating that Hx regulates extracellular free heme levels and the heme-Hx complexes protect primary neurons against the heme-induced toxicity (Li et al., 2009).

The rationale for the use of Hx as a therapeutic is based on the idea that it acts by scavenging circulating free heme, the ultimate mediator of hemoglobin toxicity. In particular, the crucial role of Hx in the protection from heme toxicity has relevance to pathological conditions associated with hemolysis that are often characterized by partial/total exhaustion of hemoglobin/heme scavengers. Therefore, replenishing the circulating stores of heme scavengers, thereby compensating for the loss of heme scavenging capacity in plasma, may be used as a therapeutic approach to target circulating free heme and prevent its deleterious effects (Schaer and Buehler, 2013; Schaer et al., 2013b; Vinchi and Tolosano, 2013). The recent demonstration of the effectiveness of an Hx therapy in mouse models of sickle cell anemia, β-thalassemia and sepsis opens new perspectives concerning the use of this molecule as a new therapeutic drug in hemolytic diseases.

To date, we are still far from the use of heme scavengers as therapeutics and no clinical trials are ongoing. Anyway, the possible use of these molecules for therapeutic purposes has elicited the interest of the research community in the field as well as of several pharmaceutical companies. Pre-clinical studies are ongoing with the aim of translating the protective effects of heme scavengers into clinical practice in the near future.

### Pathological conditions associated with alterations of heme synthesis

#### Porphyrias

Eight distinct types of inherited porphyrias have been described, each resulting from a partial deficiency of a specific enzyme of the heme biosynthetic pathway (Figure 2 and Table 2). The porphyrias are characterized by an impairment of heme synthesis, leading to the accumulation of specific intermediates of the heme biosynthetic pathway in various tissues. Depending on the primary site of overproduction and accumulation of heme precursors, the porphyrias have been traditionally classified as “hepatic” or “erythropoietic.” However, many types of porphyrias have overlapping features. The accumulation of porphyrins in different tissues leads to hepatic and hematopoietic alterations, neurological and/or cutaneous symptoms.

**Table 2:** **Disorders associated to mutations in genes involved in heme metabolism**.

**Disease name**	**Omim**	**Genetic defect**	**Disease features**
X-linked erythropoietic protoporphyria (XLEPP)	300752	*ALAS2*	*Erythropoietic Porphyria:* immediate photosensitivity; secondary liver disease; microcytic anemia
Alad-deficiency porphyria (ADP)	612740	*ALAD*	*Hepatic Porphyria:* acute neurological attacks; abdominal pain
Acute intermittent porphyria (AIP)	176000	*HMBS*	*Hepatic Porphyria:* acute neurological attacks
Congenital erythropoietic protoporphyria (CEP)	263700	*UROS*	*Erythropoietic Porphyria:* photosensitivity; hemolytic anemia
Porphyria cutaneous tarda (PCT)	176100	*UROD*	*Hepatic Porphyria:* photosensitivity; blisterin skin lesions; liver disease
Hereditary coproporphyria (HCP)	121300	*CPOX*	*Hepatic Porphyria:* acute neurological attacks; abdominal pain; vesiculoerosive skin disease photosensitivity
Porphyria variegate (VP)	176200	*PPOX*	*Hepatic Porphyria:* acute neurological attacks; abdominal pain; vesiculoerosive skin disease; neurovisceral symptoms photosensitivity
Erythropoietic protoporphyria (EPP)	177000	*FECH*	*Erythropoietic Porphyria:* photosensitivity; secondary liver disease; microcytic anemia
X-linked sideroblastic anemia (XLSA)	300751	*ALAS2*	Microcytic anemia with ringed sideroblasts
Human HO-1 Deficiency	614034	*HO1*	Hemolytic anemia, asplenia, renal and hepatic iron deposition, endothelial dysfunction/vasculitis
Posterior column ataxia and retinitis pigmentosa (PCARP)	609033	*FLVCR1*	Sensory ataxia; Retinitis Pigmentosa
Hyperuricaemia and Gout	138900	*ABCG2*	Metabolism disorder, local immune-mediated inflammatory reaction
Fowler syndrome (FS)	225790	*FLVCR2*	Cerebral glomeruloid vasculopathy; limb deformities
Hereditary Folate Malabsorption syndrome (HFM)	229050	*HCP1/PCFT*	Systemic and cerebrospinal folate deficiency; anemia; pancytopenia; hypoimmunoglobulinemia; developmental delay; cognitive impairment; epilepsy

Three different types of “erythropoietic protoporphyria” have been described. The initial accumulation of the porphyrin precursors occurs primarily in bone marrow erythroid cells. Mutations in the *FECH* gene cause erythropoietic protoporphyria (EPP; OMIM: #177000) (Gouya et al., 2002); the partial deficiency of FECH leads to the accumulation of free-PPIX in bone marrow, erythrocytes, plasma and finally in the liver (Murphy, 2003; Lecha et al., 2009). A clinically indistinguishable form of erythropoietic protoporphyria, named X-linked erythropoietic protoporphyria (XLEPP; OMIM: #300752), is due to gain of function mutations in the *ALAS2* gene (Whatley et al., 2008). The overexpression of ALAS2 causes an increased PPIX production in spite of normal FECH activity; as iron became limiting, FECH uses its alternative metal substrate leading to the accumulation of Zn-protoporphyrin in erythrocytes. The major phenotype of EPP and XLEPP is porphyrin-induced photosensitivity. Hypochromic, microcytic anemia is also common and the disorders may evolve to severe hepatobiliary disease and hepatic failure (Anstey and Hift, 2007; Whatley et al., 2008; Lecha et al., 2009). Mutations in the *UROS* gene cause the third form of erythropoietic protoporphyria, called congenital erythropoietic protoporphyria (CEP; OMIM: #263700); the partial loss of UROS activity leads to the incomplete metabolism of hydroxymethylbilane and the accumulation of non-physiologic porphyrin isomers in the bone marrow, erythrocytes, urine and other organs. CEP is mainly characterized by cutaneous photosensitivity and hemolytic anemia (Sassa and Kappas, 2000; Murphy, 2003; Bishop et al., 2006).

The “hepatic porphyrias” are characterized by the overproduction and initial accumulation of porphyrin precursors in the liver. Five different types of hepatic protoporphyria have been described. Mutations in the *ALAD, HMBS, CPOX, PPOX* genes cause different forms of acute hepatic porphyrias: Alad-deficiency porphyria (ADP; OMIM: #612740), acute intermittent porphyria (AIP; OMIM: #176000), hereditary coproporphyria (HCP; OMIM: #121300) and porphyria variegate (VP; OMIM: #176200), respectively. These disorders are characterized by the accumulation of ALA, porphobilinogen and/or porphyrins primarily in the liver. The major manifestations of these disorders are the life-threatening acute neurologic attacks and abdominal pain (Balwani and Desnick, 2012). Mutations in the *Urod* gene cause a cutaneous form of hepatic porphyria, called porphyria cutaneous tarda (PCT; OMIM: #176100); impaired *UROD* activity leads to the accumulation of uroporphyrin and other highly carboxylated porphyrins in the skin, liver and erythrocytes. PCT is characterized by blistering skin lesions that appear most commonly on the backs of the hands (increased cutaneous photosensitivity) and liver disease whereas neurologic features are usually absent (Frank and Poblete-Gutierrez, 2010; Balwani and Desnick, 2012).

The generation of animal models of porphyrias allowed a better understanding of the pathophysiological mechanisms involved in the distinct types of porphyria as well as the development of novel therapeutic strategies (Richard et al., 2008).

#### Sideroblastic anemias

Mutations in the *ALAS2* gene are responsible for the most common form of inherited sideroblastic anemia, named X-linked sideroblastic anemia (XLSA; OMIM: #300751) (Table 2). Sideroblastic anemias are genetically and clinically heterogeneous disorders characterized by the pathological accumulation of iron in the mitochondria of erythroid precursors. XLSA is due to loss of function mutations in the *ALAS2* gene (Bergmann et al., 2010; Ducamp et al., 2011) or mutations in an enhancer of *ALAS2* gene that cause the disruption of a GATA binding site important for its transcriptional regulation (Kaneko et al., 2013). The reduced activity of ALAS2 causes an impairment of heme biosynthesis; as PPIX production is decreased, excess iron accumulates in perinuclear mitochondria of erythroblasts creating a ring-like appearance and thus the characteristic “ringed sideroblasts.” XLSA patients are characterized by hypochromic, microcytic anemia of variable severity.

The role of ALAS2 in XLSA has been confirmed in several animal models of the disease. *Sauternes* (*sau*) is a zebrafish mutant characterized by a delay in erythroid differentiation, abnormal globin gene expression and heme deficiency. Using positional cloning strategies, it has been reported that the *sau* gene encodes for ALAS2, thus confirming that loss of ALAS2 leads to a microcytic, hypochromic anemia similar to XLSA (Brownlie et al., 1998). The absence of *Alas2* in mice causes embryonic lethality due to a severe block of erythroid differentiation. In contrast to human patients, ring sideroblasts are not present and iron deposition occurs in the cytoplasm instead of mitochondria (Nakajima et al., 1999; Harigae et al., 2003).

Interestingly, other forms of inherited sideroblastic anemias are due to mutations in genes indirectly involved in the heme biosynthetic pathway. Mutations in the gene coding for *SLC25A38*, the putative mitochondrial exporter of ALA, have been identified in patients with an autosomal recessive form of sideroblastic anemia (Guernsey et al., 2009). In other patients, mutations in the ATP-binding cassette transporter *ABCB7* gene or in *GLUTAREDOXIN5* (*GLRX5*) gene have been identified (Allikmets et al., 1999; Camaschella et al., 2007). Both ABCB7 and GLRX5 are involved in the assembly of iron-sulfur clusters (Lill and Muhlenhoff, 2006). Iron-sulfur clusters deficiency causes the activation of IRP1, mitochondria iron accumulation and cytosolic iron depletion that in turn activates IRP2. The activation of IRPs determines the translational repression of ALAS2 resulting in sideroblastic anemia (Allikmets et al., 1999; Wingert et al., 2005; Camaschella et al., 2007; Sheftel et al., 2009; Ye et al., 2010). Thus, a primary defect in iron-sulfur clusters biogenesis secondarily affects heme synthesis in erythroblasts, resulting in mitochondrial iron loading and the same pathophysiology of ALAS2 deficiency.

### Pathological conditions associated with alterations of heme degradation

#### Disorders associated with mutations in HO-1 gene

Mice lacking functional HO-1 were generated by Poss and Tonegawa (1997) (Table 1). These mice were characterized by serum iron deficiency and pathologic tissue iron-loading, indicating that HO-1 is crucial for the expulsion of iron from tissue stores and for its reutilization. *HO-1*^−/−^ mice were shown to accumulate, with age, hepatic and renal iron that contributed to oxidative damage, tissue injury and chronic inflammation (Yachie et al., 1999; Koizumi, 2007). These data demonstrated that, although HO-1 is a stress-induced protein, it is important under basal conditions to protect liver and kidney from oxidative damage and that it is an essential regulator of iron metabolism and homeostasis. Additionally, these mice suffered from delayed growth and progressive chronic inflammatory diseases as suggested by an enlarged spleen and lymph nodes, hepatic inflammatory cell infiltrates, vasculitis, and glomerulonephritis. Furthermore, they were found to be extremely sensitive to oxidative injury and prone to hepatic necrosis and death upon lipopolysaccharide administration.

Recent studies on *HO-1*^−/−^ mice revealed that HO-1 deficiency causes the depletion of resident splenic and liver macrophages, due to their inability to catabolize hemoglobin-derived heme during erythrophagocytosis (Kovtunovych et al., 2010). In the spleen, initial splenic enlargement was observed to progress to red pulp fibrosis, atrophy, and functional hyposplenism in older mice. Finally, the failure of tissue macrophages to remove senescent red blood cells led to intravascular hemolysis, circulating hemoglobin release, and iron redistribution to hepatocytes and kidney proximal tubules. Indeed, the lack of HO-1 strongly impairs macrophage function, thus causing iron redistribution and severe oxidative tissue injury.

HO-1 deficiency in humans was described for the first time in 1999 (OMIM: #614034) (Yachie et al., 1999; Kawashima et al., 2002) (Table 2). The sequence analysis of the *HO-1* gene revealed the complete loss of exon 2 on the maternal allele and a 2-nucleotide deletion in exon 3 on the paternal allele. The disease was reported in a 6-year-old boy, who suffered from severe growth retardation, asplenia, marked hepatomegaly, renal injury, tissue iron deposition and paradoxically elevated Hp levels. Moreover, he showed increased red blood cells fragility, chronic hemolysis, anemia, leukocytosis, thrombocytosis, disseminated intravascular coagulation, hyperlipidemia and mesangio-proliferative glomerular changes, likely resulting from endothelial injury and reticulo-endothelial dysfunction.

The serum level of Hp is usually reduced in hemolytic states; in this patient suffering from hemolytic anemia, however, the Hp level was rather increased. In the HO-1 deficient case, Hp production rather than its consumption could be increased due to a dominant effect of inflammation. In addition, reticuloendothelial dysfunction could delay the clearance of the Hp-hemoglobin complex. Contrarily to the *HO-1*^−/−^ mouse, in human HO-1 deficiency fatty streaks and fibrous plaques were observed. Histologically, the fibrous plaques were characterized by the proliferation of smooth muscle cells and few foam macrophages. The enhanced proliferation of smooth muscle cells could be a consequence of HO-1 loss.

Recently, another case of HO-1 deficiency was described in a 15-year-old girl who presented massive hemolysis, inflammation, nephritis and congenital asplenia (Radhakrishnan et al., 2011). The key features that suggested HO-1 deficiency were marked hemolysis, generalized inflammation with evidence of endothelial injury and nephropathy with underlying asplenia. Mutation analysis showed the presence of homozygous missense mutations in exon 2 (R44X) on chromosome 22q12, which resulted in the absence of the functional HO-1.

HO-1 deficiency in humans is characterized by total asplenia and this is fully recapitulated by the mouse model of HO-1 deficiency suggesting that this enzyme has a key role in macrophage heme metabolism and heme-iron reutilization. On the other hand, compared with the knockout mouse model, the human cases of HO-1 deficiency were observed to involve more severely the endothelial cells. Both the reported cases of HO-1 deficiency presented endothelial dysfunction, systemic inflammation and hemolysis. In particular, in the last reported case of HO-1 deficiency, the initial trigger of cold antibody-mediated hemolytic anemia, with intravascular hemolysis, would have resulted in heme-induced endothelial damage. In both cases, signs of severe endothelial damage in the form of raised inflammatory markers, von Willebrand factor, and coagulopathy were found and these children died of intracranial hemorrhage. It was proposed that HO-1 deficiency results in a novel form of vasculitis or endothelial injury syndrome. How asplenia modulated the disease is unexplained. In view of the absence of spleen in both cases, it is possible that HO-1 gene expression may have a role in splenic arteriogenesis, other than in angiogenesis, as already proposed.

This very rare condition provided important insight into the functional role and importance of this enzyme. In both mice and human cases, a severe proinflammatory and pro-oxidant phenotype was noted, further highlighting the anti-oxidant, anti-inflammatory and cytoprotective function of this enzyme. Although it appears evident that HO-1 is essential to keep intracellular heme levels within a physiologic non-toxic range, it is not clear whether the protection against heme toxicity by this enzyme mainly relies on its primary heme degrading activity, on the biologic activities of its metabolic end products, namely CO and bilirubin, or both.

### Disorders influenced by HO-1 polymorphisms

Humans differ quantitatively in their ability to build up an HO-1 response and variations in HO-1 expression dictate the pathologic outcome of a broad range of diseases. This differential response seems to be modulated by two polymorphisms in the HO-1 gene promoter region. Several studies demonstrated that the ability of a patient to respond strongly in terms of upregulating HO-1 may be an important endogenous protective factor.

A well described (GT)n microsatellite polymorphism in the HO-1 promoter is thought to regulate the extension of HO-1 induction as well as its response to many stimuli. Individuals with a lower number of (GT)n repeats retain the ability to more efficiently induce HO-1 than individuals with a higher number of repeats. Multiple studies demonstrate that individuals with fewer repeats are less prone to certain pathologies. It is interesting to observe that most of the pathologies affected by this polymorphism in humans overlap with those in which the outcome is exacerbated by HO-1 deletion (e.g., endotoxin shock, severe sepsis, atherosclerosis, myocardial infarction, ischemia reperfusion injury). Additionally, one study correlated the presence of the shorter (GT)n polymorphism with a longer life of the individual. Individuals who lived longer were more likely to have the “high transcriptional induction” genotype, associated with the short HO polymorphism (Kimpara et al., 1997; Hirai et al., 2003; Denschlag et al., 2004; Exner et al., 2004).

Studies comparing the outcome of several experimental pathologic conditions in *HO-1*^−/−^ or wild-type mice show that when HO-1 is lacking, the pathologic conditions studied are exacerbated and result in high incidence of mortality. Pharmacological inhibition of HO activity mimics the phenotypes associated with HO-1 deletion, while, on the other hand, induction of HO-1 or administration of its reaction products, CO and biliverdin, is protective and usually ameliorates the pathologic conditions. The cytoprotective effects of HO-1 and/or of its final products, CO and biliverdin/bilirubin have been demonstrated in a variety of diseases, including immune-mediated inflammatory diseases (rejection of transplanted organs, autoimmune diseases, asthma, arthritis, colitis, pancreatitis, recurrent abortions), infectious diseases (severe malaria, sepsis, endotoxin shock), cardiovascular diseases (atherosclerosis, myocardial infarction, endothelial dysfunction, vaso-occlusion) and ischemia-reperfusion injury (Gozzelino et al., 2010).

Several molecules that exert a beneficial effect against different diseases are known to act via HO-1 induction. Some of these synthetic compounds include sialic acid, statins and rapamycin. In addition, several molecules produced under physiologic conditions might act in a similar manner, including the anti-inflammatory cytokine interleukin (IL)-10, some prostaglandins, VEGF (vascular endothelial growth factor), stromal cell-derived factor 1, nitric oxide (NO) and NGF (nerve growth factor) (Gozzelino et al., 2010). The mechanisms via which these molecules could induce the expression of HO-1 is the inhibition of Bach1 activity and the activation of transcription factors that promote HO-1 transcription, such as Nrf2. Alternatively, therapeutic induction of endogenous HO-1 might be obtained via the delivery of non-cytotoxic amount of heme or heme-containing proteins, the so-called preconditioning. In this manner, the administration of hemoglobin or heme was shown to improve survival in response to endotoxic shock (Otterbein et al., 1995), to reduce liver and kidney injury (Nath et al., 1992) and decrease vascular stasis (Belcher et al., 2006, 2010). Similarly, the preconditioning of Hx^−/−^ mice with a small dose of hemin strongly decreases their susceptibility to acute heme overload, thus limiting heme-mediated tissue damage (Vinchi et al., 2008). Interestingly, a physiological paradigm of heme preconditioning is represented by sickle cell trait, in the heterozygous form. This mutation confers protection against severe forms of malaria due to the low concentrations of heme in the circulation that lead to induction of endogenous HO-1 expression and protection against *Plasmodium* infection (Ferreira et al., 2011). In this condition, chronic hemolysis associated to sickle cell disease induces a state of malaria tolerance by inducing HO-1 up-regulation, which directly supports heme degradation and drives the generation of anti-inflammatory and anti-oxidant metabolites. Additionally, the ability of CO to directly bind hemoglobin and inhibit its oxidation, thus avoiding heme release from oxidized hemoglobin, has been recently shown to prevent experimental cerebral malaria, thus highlighting a new and key mechanism of HO-mediated protection in pathologies associated with hemoglobin/heme release (Pamplona et al., 2007; Ferreira et al., 2008; Seixas et al., 2009).

Clinical trials based on the use of CO are currently ongoing to evaluate the potential of CO as a therapeutic agent in humans. CO is administered by inhalation for the treatment of pathologies such as pulmonary arterial hypertension, post-operative ileus and idiopathic pulmonary fibrosis (see http://www.clinicaltrials.gov website). Alternatively, in the last decade a great effort was made to design and study molecules able to bind and deliver CO in biological systems, known as CO-releasing molecules (CO-RMs) (Motterlini et al., 2005). In the next years the evaluation of the therapeutic potential of CO via inhalation or CO-RM administration will reveal whether this way may be pursued as a therapeutic strategy to counteract pathological outcome due to heme-driven toxicity.

## Pathological conditions associated with mutations in genes coding for heme transporters

### Posterior column ataxia and retinitis pigmentosa (PCARP)

Recently, *FLVCR1* has been reported to be the causative gene for PCARP (OMIM: #609033) (Rajadhyaksha et al., 2010; Ishiura et al., 2011) (Table 2). PCARP is a childhood-onset, autosomal-recessive, neurodegenerative syndrome with the clinical features of sensory ataxia and retinitis pigmentosa. PCARP begins in infancy with areflexia and retinitis pigmentosa. During infancy, night blindness, peripheral visual loss with subsequent progressive constriction of the visual field and loss of central retinal function became apparent. The sensory ataxia caused by the degeneration of the posterior column of the spinal cord results in a loss of proprioceptive sensation. Scoliosis, camptodactyly, achalasia, gastrointestinal dysmothility and a sensory peripheral neuropathy are variable features of the disease. PCARP is considered a sensory ganglionopathy causing a degeneration of central and peripheral axons without evidence of primary demyelination (Higgins et al., 1997).

Four different homozygous mutations in *FLVCR1* gene have been reported to date. Interestingly, three of these mutations (c.361A>G, c.574T>C, and c.721G>A) (Rajadhyaksha et al., 2010) occur in the first exon of *FLVCR1* gene, thus probably affecting only FLVCR1a. However, we cannot exclude the possibility that these mutations could interfere with any still unknown regulatory sequences important for FLVCR1b expression. The other mutation (c1477G>C) (Ishiura et al., 2011) is in the tenth exon of *FLVCR1* gene, common to both *Flvcr1a* and *Flvcr1b* transcripts (Chiabrando et al., 2012), thus probably affecting both transporters. Each mutation corresponds to a specific aminoacid substitution (Asn121Asp, Cys192Arg, Ala241Thr, and Gly493Arg) within putative transmembrane domains of FLVCR1: 1, 3, 5, and 12 transmembrane domains, respectively. Yanatori et al. (2012) investigated the consequences of the reported *FLVCR1* mutations on the plasma membrane heme exporter FLVCR1a. *In vitro* studies indicate that all the four identified mutations affect the subcellular localization, half-life and heme-export function of FLVCR1a (Yanatori et al., 2012). However, the consequences of all these mutations on the expression, localization and activity of FLVCR1b have not been investigated yet. It has been proposed that the mutant FLVCR1a proteins fail to fold properly in the endoplasmic reticulum and are rapidly degraded in the lysosomes; loss of FLVCR1a could lead to heme overload, oxidative stress and apoptosis of photoreceptors in the retina and sensory neurons in the posterior column of the spinal cord of PCARP patients (Yanatori et al., 2012). Actually, this is merely a hypothesis and evidences of heme overload, heme-induced toxicity, and apoptosis in PCARP patients or mouse models of the disease are still lacking. Of note, apoptosis is the final and common cause of photoreceptors degeneration in several forms of retinitis pigmentosa (Marigo, 2007).

The reason why the loss of FLVCR1a could affect two specific sensory modalities, vision and proprioception, is unknown. The expression levels of *Flvcr1* mRNA have been confirmed in the mouse brain (neocortex, striatum, hippocampus, and cerebellum), posterior column of the spinal cord, retina and retinal pigment epithelium. The highest levels of *Flvcr1* mRNA have been found in the retina and spinal cord thus suggesting a correlation between the regional specificity of *Flvcr1* mRNA expression and the selective degenerative pathology of PCARP (Rajadhyaksha et al., 2010; Gnana-Prakasam et al., 2011). However, FLVCR1 is ubiquitously expressed (Quigley et al., 2004; Chiabrando et al., 2012) and the observation that mutations in the *FLVCR1* gene cause sensory ataxia and retinitis pigmentosa was completely unexpected. Two different mouse models of FLVCR1 deficiency have been generated; in both cases, the loss of specific FLVCR1 isoforms severely affects embryonic development (Keel et al., 2008; Chiabrando et al., 2012), a stronger phenotype compared to that of PCARP patients. Furthermore, the mouse models of *Flvcr1* deficiency suggest an essential role of FLVCR1 isoforms in erythroid differentiation and in the maintenance of endothelial integrity (Keel et al., 2008; Chiabrando et al., 2012). PCARP patients did not suffer from anemia (Rajadhyaksha et al., 2010; Yanatori et al., 2012). On the other hand, neurological defects have not been reported in mouse models of *Flvcr1* deficiency. The reasons of the discrepancies between the human disease and the mouse models need to be further investigated.

Further work is needed to understand how the loss of a ubiquitously expressed heme exporter could lead to the selective pathological features of PCARP.

### Diamond blackfan anemia (DBA)

A role for *FLVCR1* in the pathogenesis of Diamond Blackfan Anemia (DBA; OMIM: #105650) has been proposed due to the observation that the feline leukemia virus subgroup C causes a pure cell aplasia in cats by interfering with the expression of FLVCR1 and that mice lacking both FLVCR1 isoforms phenocopy the human disease (Tailor et al., 1999; Quigley et al., 2000; Keel et al., 2008).

DBA is an autosomal dominant disorder with incomplete penetrance due to mutations in several genes coding for ribosomal proteins; the most common mutation is in ribosomal protein S19 gene (RPS19) (Campagnoli et al., 2008). DBA patients are characterized by a severe block of erythroid differentiation at the proerythroblast stage associated to congenital malformations and cancer predisposition (Flygare and Karlsson, 2007; Chiabrando and Tolosano, 2010; Narla and Ebert, 2010). Accordingly, FLVCR1 deficient mice are characterized by a block of erythroid differentiation as well as growth defects (Keel et al., 2008).

Mutations in *FLVCR1* gene have not been identified in DBA patients (Quigley et al., 2005). Rey et al. (2008) identified several alternatively spliced *Flvcr1* transcripts, encoding proteins with aberrant expression and function. Due to the experimental strategy, only *Flvcr1a* transcript was evaluated in this work. Interestingly, the aberrant alternative splicing of *Flvcr1a* is increased in immature erythroid cells of some DBA patients negative for RPS19 mutations, while the expression of the wild-type protein is decreased (Rey et al., 2008). The molecular mechanism leading to the aberrant alternative splicing of *Flvcr1a* in DBA is unknown. These data suggest that decreased FLVCR1a expression and alteration of heme metabolism could contribute to the pathogenesis of DBA.

The identification of FLVCR1b essential role in heme synthesis and erythroid differentiation, leads to the hypothesis that alteration of FLVCR1b expression could also concur to the pathogenesis of DBA (Chiabrando et al., 2012). Whether aberrant alternative splicing of *Flvcr1b* transcript also occurs in DBA immature erythroid cells, still needs to be addressed.

Mouse models of *Flvcr1* deficiency are also characterized by defective growth (Keel et al., 2008) and skeletal malformations (Chiabrando et al., 2012). Thus, FLVCR1a could also have a role in the development of the congenital malformations observed in DBA patients.

Although *FLVCR1* is far to be the causative gene for DBA, we could speculate that the aberrant expression of FLVCR1 isoforms could contribute to different pathological features of DBA.

Considering the essential role of FLVCR1 isoforms during erythroid differentiation, it will be interesting to analyze their expression also in other erythroid diseases. An alteration of the expression of FLVCR1 isoforms may play a role in the pathogenesis of disorders characterized by an imbalance between heme and globin synthesis too.

### Putative role of FLVCR1 in drug metabolism

The recent finding that liver conditional *Flvcr1a*^−/−^ mice show a reduced expression and activity of cytochromes P450 suggests that heme export may have an impact on cytochrome function. About 50% of hepatic heme is used for synthesis of P450 enzymes, which metabolize exogenous and endogenous compounds, including hormones, xenobiotics, drugs, and carcinogens. It is well-known that a reduction in heme availability due to an enhanced heme degradation leads to the impairment of cytochrome function (Correia et al., 2011). On the other hand, the lack of FLVCR1a-mediated heme export in hepatocytes, although leading to an increase in the cytosolic heme pool, similarly causes a reduction in cytochrome activity. In hepatocytes, heme is likely formed in excess over its metabolic needs and heme homeostasis is ensured by a combination of synthetic, degradative, and export mechanisms. FLVCR1a, by exporting heme excess out of the cell, controls the size of the cytosolic heme pool, thus allowing proper heme synthesis and new cytochrome formation. FLVCR1a loss causes heme synthesis inhibition, due to the expansion of the cytosolic heme pool, ultimately leading to a decrease in cytochrome function (Vinchi et al., 2014). These observations have potential implications for hepatic metabolism of xenobiotics and drugs. In particular, deletion or mutations in *Flvcr1a* and other pathologic conditions that reduce its expression potentially cause a reduction in cytochrome activity, thus altering drug metabolism. Therefore, individuals that routinely assume drugs for therapeutic purposes may show a reduced ability to induce cytochromes P450 and to metabolize pharmaceuticals, thus being more susceptible to drug intoxication. In the near future, the mouse model of FLVCR1a deletion in hepatocytes could be applied for metabolic studies to address the importance of hepatic heme export upon drug administration.

### Hyperuricaemia and gout

As stated above, ABCG2 was initially shown to transport a wide range of substrates including drugs, xenobiotics, food metabolites and heme. Only recently, genetic studies in humans have identified urate as a physiological substrate of ABCG2, perhaps the most relevant. Indeed, a genome-wide association study identified common alleles in *ABCG2* as associated with serum urate level and risk of gout (OMIM: #138900) (Table 2) (Dehghan et al., 2008). ABCG2 expression in the apical membrane of human proximal tubule cells (Doyle and Ross, 2003), the main site of urate handling in the kidney, is consistent with a role for ABCG2 in urate excretion. Gout is a common form of inflammatory arthritis and occurs when uric acid crystallizes in the form of monosodium urate, precipitating in joints, on tendons, and in the surrounding tissues. These crystals then trigger a local immune-mediated inflammatory reaction. Genetic studies identified a SNP in the exon 5 of *ABCG2* gene that leads to a glutamine-to-lysine amino acid substitution (Q141K) (Dehghan et al., 2008). The Q141K variant has been shown to have a significant reduced capacity to transport urate and, when expressed in mammalian cells, Q141K ABCG2 expression is significantly lower than that of wild-type protein (Imai et al., 2002; Mizuarai et al., 2004; Morisaki et al., 2005; Woodward et al., 2009). Another study on the Japanese population identified a different variant as associated to hyperuricemia and gout (Yamagishi et al., 2010).

Many additional SNPs in *ABCG2* gene have been identified and associated to altered drug resistance (Woodward et al., 2011). Future studies will have to address whether additional SNPs also affect serum urate concentration in human. Finally, how these polymorphisms affect heme transport remains to be determined.

### Fowler syndrome

Mutations in the *FLVCR2* gene have been associated to the Fowler syndrome (OMIM: #225790) (Table 2), a rare lethal autosomal-recessive disorder of brain angiogenesis, first described in 1972 (Fowler et al., 1972). The Fowler syndrome is a cerebral proliferative glomeruloid vasculopathy resulting in abnormally thickened and aberrant perforating vessels, forming glomeruloid structures throughout the central nervous system. These defects lead to hydranencephaly and hydrocephaly and are restricted to the central nervous system parenchyme. In addition to cerebral proliferative glomeruloid vasculopathy, fetuses affected by the Fowler syndrome also show limb deformities.

Several kinds of mutations were identified in cases of Fowler syndrome (Meyer et al., 2010; Thomas et al., 2010), but it is not clear how these mutations could affect *FLVCR2* expression, functions or localization.

The mechanism by which *FLVCR2* mutations could lead to abnormal angiogenesis is also unknown. Some hypotheses have been put forward.

First, it has been postulated that the role of FLVCR2 in calcium trafficking, proposed by Brasier et al. (2004), could explain its involvement in Fowler syndrome. Indeed, the altered angiogenesis in Fowler syndrome has been supposed to be due to a deficit in pericytes, cells essential for capillary stabilization and remodeling during brain angiogenesis (Thomas et al., 2010). These cells interact with endothelial cells, whose proliferation and motility are calcium-dependent events. Moreover, the Fowler syndrome has been associated with varying degrees of calcification throughout the central nervous system (Harding et al., 1995).

Although possible, this hypothesis seems to be inadequate because the involvement of FLVCR2 in calcium transport has not been demonstrated. Moreover, it is not clear whether the effects on pericytes observed in fetuses affected by Fowler syndrome is the primary cause or an effect of the disease.

A second hypothesis to explain *FLVCR2* implication in the Fowler syndrome is based on its heme import activity. It has been observed in some cases of Fowler syndrome that there is a deficiency in the heme-dependent complexes IV of the mitochondrial electron transport chain, essential for the production of adenosine triphosphate by oxidative phosphorylation. Since young neurons that migrate to the neocortex are dependent on oxidative phosphorylation, it has been speculated that *Flvcr2* disruption could lead to dysfunction of the mitochondrial electron transport chain, thus causing the neurodegeneration and the developmental abnormalities found in Fowler syndrome (Duffy et al., 2010).

Finally, a third hypothesis is that alteration on FLVCR2 heme-import activity could account for cellular iron overload, a condition known to be associated to neurodegeneration and bone abnormalities (Duffy et al., 2010).

Although all conceivable, the three hypotheses need experimental verification and it remains unclear why the damaged areas in Fowler syndrome are restricted to the central nervous system and whether the proliferative vasculopathy is primary or secondary to the neurodegeneration.

### Hereditary folate malabsorption syndrome

The only known disorder associated to mutations in the *HCP1/PCFT* gene relates to its role in the transport of folates, while the effects of the loss of its heme-import functions seem to be negligible, suggesting the instauration of alternative compensatory mechanisms to support cellular heme-iron uptake in the absence of HCP1/PCFT.

Mutations in the *HCP1/PCFT* gene have been associated to the hereditary folate malabsorption syndrome (HFM; OMIM: #229050), a rare autosomal recessive disorder first described in 1961 (Luhby et al., 1961) (Table 2).

Folates are essential cofactors required for key epigenetic and biosynthetic processes, such as de novo synthesis of purines and pyrimidines, methionine and deoxythymidylate monophosphate. Mammals do not synthesize folates, and body needs are met by duodenal and jejunal absorption of folates contained in the diet.

The basis for HFM is the impaired intestinal folate absorption, resulting in severe systemic and cerebrospinal fluid folate deficiency in a few months after birth.

Patients affected by HFM show anemia, diarrhea, pancytopenia, hypoimmunoglobulinemia, and frequent neurological dysfunctions like developmental delays, cognitive impairment, and epilepsy. Many kinds of mutations have been reported (Qiu et al., 2006; Zhao et al., 2007; Shin et al., 2011; Diop-Bove et al., 2013), occurring in different sites of *HCP1/PCFT* gene and with different effects on the protein encoded by the mutated alleles.

If untreated, the disease is fatal and, if treatment is delayed, the neurologic deficits can become permanent. Normalization of blood, and rarely cerebrospinal fluid, folate level could be achieved by administration of low doses of parenteral folates or of pharmacological doses of oral folates. In the latter case, folates are probably absorbed by the bidirectional Reduced Folate Carrier 1 (RFC1), a folate importer alternative to HCP1/PCFT, which is expressed in the upper small intestine. RFC1 shows a lower affinity for folates as compared with HCP1/PCFT, but can compensate for HCP1/PCFT loss in HFM patients treated with high doses of folates (Qiu et al., 2006; Zhao et al., 2007).

The *Hcp1/Pcft*^−/−^ mouse represents the murine model for this HFM syndrome (Salojin et al., 2011) (Table 1). *Hcp1/Pcft*^−/−^ mice die by 10–12 weeks of age and show a haematological profile which closely resembles that observed in human HFM patients.

## Concluding remarks

The excess of free-heme is toxic due to its high reactivity and its ability to promote oxidative stress on lipids, proteins and nucleic acids, eventually leading to cell death.

The existence of multiple levels of heme control indicates that mammals specifically evolved systems able to limit heme amount outside and inside the cell to counteract heme potential toxicity.

As a primary defence mechanism, Hx scavenges free circulating heme, thereby avoiding it to exert its pro-oxidant effects. Indeed, Hx saturation observed in hemolytic disorders leads to heme-driven tissue damage and oxidative stress.

The control of heme synthesis and degradation has a key role in the regulation of intracellular heme levels and these two mechanisms are respectively inhibited and induced by heme itself. Alterations in these processes lead to pathological iron or heme accumulation. Mutations in *ALAS2* gene cause insufficient heme production, mitochondria iron overload, and oxidative stress, as observed in sideroblastic anemia (Sheftel et al., 2009). HO-1 deficiency leads as well to anemia and inability to efficiently reuse tissue heme-iron. Moreover, HO-1 has a cytoprotective action that resides not only in the degradation of toxic heme but also in the production of the antioxidant molecules CO and biliverdin.

The recent identification of plasma membrane heme transporters suggests that intracellular heme amount could be regulated not only at the level of its synthesis and catabolism but also at the level of heme export and import. Nevertheless, apart FLVCR1 for which studies in mouse models have provided compelling evidence that it exerts a physiologic role as a heme exporter, the experimental evidence that ABCG2, FLVCR2, and HCP1/PCFT are heme transporter with a physiologic role in heme/iron homeostasis are very poor. Thus, it would be more correct to refer to these molecules as secondary regulators of heme and/or porphyrin metabolism. This conclusion is corroborated by genetic studies in humans that failed in finding a clear association between mutations in genes coding for heme transporters and disorders of heme metabolism as was expected from studies on cellular and animal models. Mutations in *FLVCR1* or *FLVCR2* affect specific cell populations, sensory neurons and photoreceptors or vascular cells of the central nervous system, respectively. Polimorphisms in *ABCG2* result in defective urate excretion while mutations in *HCP1/PCFT* cause folate malabsorption. These discrepancies may be related to the different kind of mutations taken into considerations, loss-of-function mutations in mice and point mutations resulting in proteins with altered function/localization in humans. It is possible that null mutations can affect heme metabolism also in humans or that specific polimorphisms in genes coding for heme transporters can differentially alter the import/export of specific substrates. The full comprehension of the role of FLVCR1-mediated heme export in human physiology and pathology is a challenge for future studies as well as the definition of the role of FLVCR2, ABCG2, and HCP1/PCFT in heme metabolism. Of course, the definition of protein structure and the identification of residues critical for heme transport in heme transporters are very important issues. In addition, the description of the regulation of these transporters in different tissues and following different stimuli will be of relevance to fully understand the multi-level system of heme control.

In the future, the comprehension of these mechanisms could have important clinical implications. Targeting heme transporters could be therapeutically useful to prevent heme-driven tissue damage in pathologies characterized by enhanced oxidative-stress.

### Conflict of interest statement

The authors declare that the research was conducted in the absence of any commercial or financial relationships that could be construed as a potential conflict of interest.
